# Metal Chelates of Sulfafurazole Azo Dye Derivative: Synthesis, Structure Affirmation, Antimicrobial, Antitumor, DNA Binding, and Molecular Docking Simulation

**DOI:** 10.1155/2023/2239976

**Published:** 2023-04-22

**Authors:** Hoda A. El-Ghamry, Rajaa O. Al-Ziyadi, Fatmah M. Alkhatib, Khadiga M. Takroni, Abdalla M. Khedr

**Affiliations:** ^1^Chemistry Department, Faculty of Applied Science, Umm Al-Qura University, Makkah, Saudi Arabia; ^2^Chemistry Department, Faculty of Science, Tanta University, Tanta, Egypt

## Abstract

A series of divalent and one trivalent metal chelates of the azo ligand resulting from coupling of sulfafurazole diazonium chloride with resorcinol have been designed and synthesized. Structure investigation of the isolated chelates have been achieved by applying spectroscopic and analytical tools which collaborated to assure the formation of the metal chelates in the molar ratios of 1L: 1M for Ni(II), Co(II), and Fe(III) chelates, where Cu(II) and Zn(II) complexes formed in the ratio 2L : 1M. The geometrical arrangement around the metal canters was concluded from UV-Vis spectra to be octahedral for all metal chelates. The attachment of the ligand to the metal ions took place through the azo group nitrogen and o-hydroxyl oxygen through proton displacement leading to the ligand being in monobasic bidentate binding mode. Antimicrobial and antitumor activities of the interested compounds have been evaluated against alternative microorganisms and cancer cells, respectively, in a trial to investigate their extent of activity in addition to docking studies. The mode of interaction of the compounds with SS-DNA has been examined by UV-Vis spectra and viscosity studies.

## 1. Introduction

The class, sulfonamide, which is produced by switching out various functional groups, can exhibit a broad range of diverse therapeutic and pharmacological vitalities [1–4]. The staggering number of publications has focused on sulfonamide complexes due to the strong propensity of sulfonamides to ligate metal ions in a variety of ways [5–8]. The tendency of sulfonamides to function as ligands is thought to be caused by the –SO_2_-NH site's acidic activity, which leads to the development of anionic donors reinforced by having O, S, and/or N atoms. The stereochemical requirements for various configurations, such as monomeric, dimeric, and polymeric structures, are met by such theme settings [9, 10]. In addition, the aromatic amino group, which is the other basic part of sulfonamides, is responsible for the chemical polarity of such compounds since it can function as an excellent coordination centre. However, what appears more significant is that this moiety is a reactive site through which sulfonamides can be largely modified generating ligands to obtain a vast number of complexes with biological importance [11, 12].

The sulfonamide compounds' azo dye derivatives are also useful as possible ligands for a variety of metal ions [13–16]. The antimicrobials made of azo sulfonamides were the first effective chemotherapeutic medications that could be administered systemically to treat bacterial infections in humans. A class of azo dye stuffs with a sulfonamide functional group is common. These azo sulfonamide dyes were developed as potential antibacterial medications [13]. Furthermore, heterocyclic azo dyes have been crucial to the improvement of the branch coordination chemistry. These substances' significance may stem from their biological potency and analytical uses [17]. Their formed metal complexes demonstrated fantastic applications in biological systems and the dying process [18, 19]. Analytical detection of several metallic elements in actual samples has been also performed using such compounds [20, 21]. Moreover, the contribution of such class of compounds in spectroscopic studies and investigation of azo dyes and their metal complexes is considerable [5, 13, 15–22].

Since cellular DNA and proteins are practically all bioactive medicines' extreme targets, the step that follows the production of a new compound intended for biological applications is to examine its activity in DNA and protein binding [22]. Furthermore, the method by which compounds bind to DNA may be important for developing new and better medications that can recognize a particular DNA location [23]. It is possible for compounds including metal complexes to bind to DNA covalently (by substituting a labile ligand in the structure of metal chelates with a nitrogen base of DNA such as guanine N7), through noncovalent bonds (by intercalation, electrostatic, or groove binding), or by a combination of the two types [24, 25]. For the intercalation mode of binding, there are two main modes, classical and threading intercalation. Classical intercalators are those compounds which attach to duplexes of DNA basically through all of their aromatic system implemented between GpG base pairs form the intercalation site top and bottom. On the other hand, in threading intercalators, the minor and major grooves of DNA are simultaneously occupied by the intercalators. Metal chelates which interact with DNA via intercalation can form both classes [26].

Preparation of metal complexes (as new drugs) in the nanometre form displays great importance in increasing the efficiency of drug absorption by human cells while minimizing the amount of required active material (i.e., less amount and higher absorption), which in turn reduces additives, medicines, and also reduces the side effects of chemical additives [27]. So, based on the previous facts, the main theme of this research is the synthesis of novel metal chelates of the sulfonamide azo ligand that is obtained by coupling of sulfafurazole diazonium chloride with resorcinol. The structures, mode of bonding, and geometry of the synthesized complexes have been affirmed using the analytical and spectral tools in addition to X-ray and TEM analysis to assign their morphology and particle size range. Bioassay evaluation of the synthesized compounds against alternative types of bacteria, fungi, and the tumor cell lines A-549 (human lung carcinoma cancer cell line) and Panc-1 (pancreatic carcinoma) have been also assured with theoretical calculations applying molecular docking simulation. Electronic absorption titration and viscosity measurements have been used to assign how the obtained compounds bind to SS-DNA.

## 2. Experimental

### 2.1. Synthesis of Sulfafurazole Azo Dye Ligand (H_3_PIBS)

**Figure 1:**
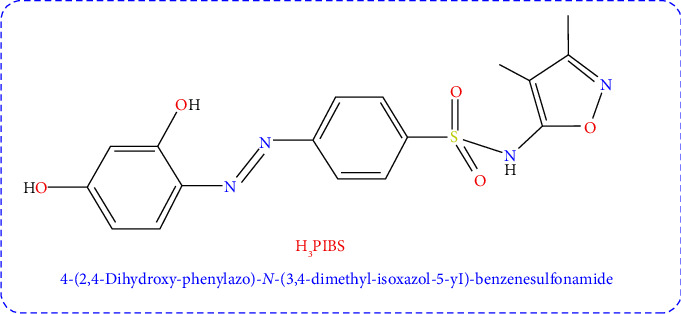
Structure and abbreviation of the ligand in interest.

The interested ligand termed 4-(2,4-Dihydroxy-phenylazo)-N-(3,4-dimethyl-isoxazol-5-yl)-benzenesulfonamide (**H**_**3**_**PIBS**) has been synthesized as cited previously [28]. The formation of the **H**_**3**_**PIBS** ligand with the predicted structure shown in Figure 1 has been confirmed by means of ^1^H-NMR, mass, and FTIR spectra.

### 2.2. Synthesis of H_3_PIBS Metal Chelates

Cu(II), Ni(II), Co(II), Fe(III), and Zn(II) chelates of **H**_**3**_**PIBS** ligand have been isolated following the next procedures:

1 mmol of CuCl_2_·2H_2_O (0.17 g), NiCl_2_·6H_2_O (0.237 g), CoCl_2_·6H_2_O (0.236 g), FeCl_3_ (0.162 g), and Zn(NO_3_)_2_·6H_2_O (0.297 g) dissolved in 15 mL of hot MeOH were separately and slowly poured into 30 mL solution hot methanolic containing 1 mmol of **H**_**3**_**PIBS** ligand (3.88 g) and 3 drops of the alkaline triethylamine. The formed mixtures were heated with reflux for nearly 2 hours within which dark products were noticed clearly. Filtration was used to separate the generated products, and then methanol and ether were used for their washing. The precipitates were then desiccated over dehydrated CaCl_2_ in a vacuum. Thin layer chromatography (TLC) was used to verify the purity of the metal chelate that was produced.

### 2.3. Analytical, Physical Methods and Instrumentations

All of the chemicals utilized, which are analytical grade and used directly without undergoing purification, were provided by Sigma-Aldrich Company. All data about the tools and methods used to confirm the compounds' structures are provided in the supplementary file (Section S1).

### 2.4. Antibacterial and Antifungal Evaluation

Antimicrobial and antifungal evaluation of the target complexes and their ligand have been carried out contra the strains *Staphylococcus aureus* (*S. aureus*), *Bacillus cereus* (*B. cereus*), *Escherichia coli* (*E. coli*), *Salmonella typhi* (*S. typhi*), *Aspergillus flavus* (*A. flavus*), and *Candida albicans* (*C. albicans*) using the well diffusion method [29]. The applied procedures in detailed are shown in Section S2 (supplementary file).

### 2.5. Cytotoxic Evaluation

The target chelates and their ligand have been alsoexamined for their cytotoxicity assay against the human lung cancer cell lines (A-549) and pancreatic carcinoma (Panc-1) applying the reported method [30, 31], which have been presented in detail in Section S3 (supplementary file).

### 2.6. Molecular Docking Simulation

The software used for docking simulation is MOE-Dock 2014 [32]. The proteins which have been applied for such study are receptors of *Staphylococcus aureus* adhesion protein (PDB ID: 4m01) in addition to caspase-3 (PDB ID: 2XYG). Docking studies details are shown in supplementary data (Section S4).

### 2.7. DNA-Binding Modes

To assess the DNA-binding propensity of the compounds of interest in the current study, salmon sperm DNA (SS-DNA) was used. Spectroscopic analysis was used to confirm that there are no proteins involved in the DNA structure. The ratio of absorbance at wavelengths 260 and 280 nm (A260/A280) has been determined to be in the range 1.8-1.9 [33]. Absorption spectroscopy and viscosity measurements have been employed as the investigative tools for determining how the compounds of interest bind to DNA.  Section S5 of the supplementary file contains details about the experiment's procedures.

## 3. Results and Discussion

### 3.1. ^1^H-NMR Spectra

**Figure 2:**
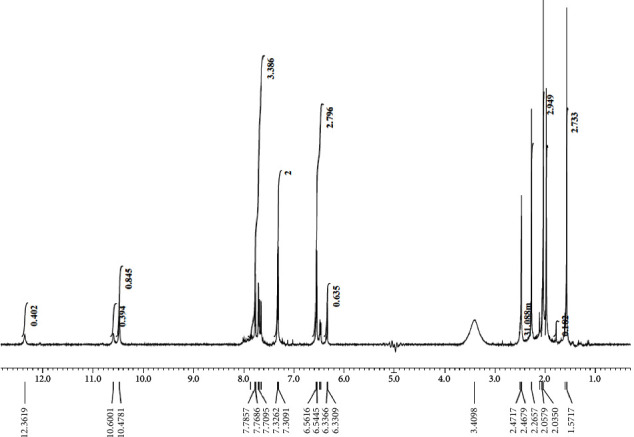
^1^H-NMR spectra of **H**_**3**_**PIBS** in d^6^-DMSO.

The ligand's, **H**_**3**_**PIBS**, ^1^H-NMR spectra were recorded in d^6^-DMSO and in the presence of D_2_O using the internal standard tetramethylsilane (TMS) (Figures 2 and S1). The main spectrum recorded in d^6^-DMSO have three singlet signals at 12.36,10.60, and 10.47 corresponding to the two hydroxide protons and NH protons, respectively [33]. These three signals are entirely missed by addition of D_2_O assuring. The eight aromatic protons appeared in the range 7.78 − 6.33 ppm. The two methyl protons with their 6 hydrogen atoms appeared as sharp singlet signals at 1.57 (attached to the C=C group) and 2.05 ppm (attached to C=N) [34].

### 3.2. Molar Conductivity and Complexes' Stoichiometry

The azo ligand **H**_**3**_**PIBS** and its five complexes, **H**_**2**_**PIBS-Cu**, **H**_**2**_**PIBS-Ni**, **H**_**2**_**PIBS-Co**, **H**_**2**_**PIBS-Fe**, and **H**_**2**_**PIBS-Zn**, afforded elemental analysis results that are remarkably comparable with the ascribed molecular formulae. Although **H**_**2**_**PIBS-Cu** and **H**_**2**_**PIBS-Zn** chelates have been synthesized in the molar ratio 1 : 1 (M : L), as illustrated in Table 1, the obtained analytical results confirmed the formation of these complexes in the molar ratio 1 : 2 (M : L). The rest of compounds have been confirmed to be constituted in the molar ratio 1 : 1. The information received from molar conductance measurement using a concentration of 10^−3^ M in DMF for each chelate showed molar conductance values inside the range 25.1–33.9 Ω^−1^·cm^2^·mol^−1^ which are matching with nonelectrolytic metal chelates [35]. The metal chelates have been confirmed to be having the empirical formulae [Cu(H_2_PIBS)_2_(H_2_O)_2_]·1.5H_2_O, [Ni(H_2_PIBS)Cl(H_2_O)_3_], [Co(H_2_PIBS)Cl(H_2_O)_3_], [Fe(H_2_PIBS)Cl_2_(H_2_O)_2_], and [Zn(H_2_PIBS) (NO_3_) (H_2_O)_2_] affording high stability in air without any colour change. All the compounds are largely soluble in high polarity solvents as DMF and DMSO but hard to dissolve or insoluble in nonpolar or low polarity solvents.

### 3.3. EI-Mass Spectra

**Figure 3:**
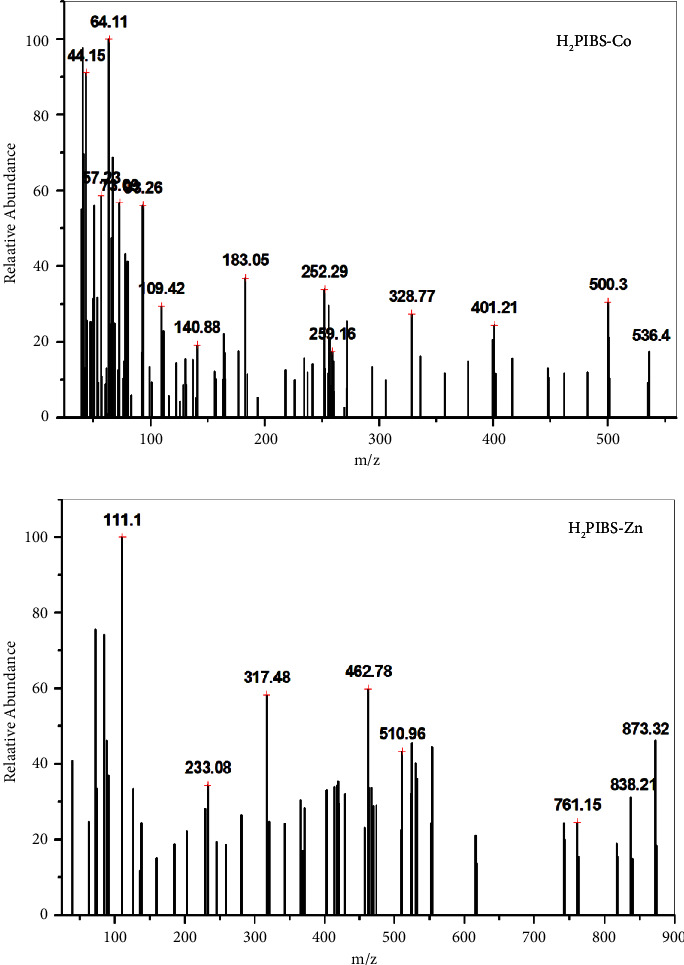
Mass spectra of **H**_**2**_**PIBS-Co** and **H**_**2**_**PIBS-Zn**.

The assignment of the molecular weight of **H**_**3**_**PIBS** ligand and its chelates has been concluded from the mass spectral investigation. As interpreted in Figures 3 and S2–S6, the molecular ion peak of **H**_**3**_**PIBS** and its complexes are obvious at *m*/*z* equal 338.02, 874.07, 535.53, 536.40, 550.84, and 873.32 which agree with the proposed theoretical values of *m*/*z* that equal 338.40, 874.38 (excluding the lattice water), 535.58, 535.82, 550.17, and 876.20 for **H**_**3**_**PIBS, H**_**2**_**PIBS-Cu**, **H**_**2**_**PIBS-Ni**, **H**_**2**_**PIBS-Co**, **H**_**2**_**PIBS-Fe**, and **H**_**2**_**PIBS-Zn**, respectively.

### 3.4. FTIR and Binding Mode Assignment

By matching the FTIR spectra of the five synthesized metal complexes with that of **H**_**3**_**PIBS**, it is possible to identify the coordinating function groups in the ligand to the metal canters. Coordinating function groups frequently exhibit changes in the position and/or intensity. Upon chelation, it is also possible for some bands to vanish.  Table 2 lists the most significant infrared bands of **H**_**3**_**PIBS** and its complexes together with their assigned values.

According to the data shown in Table 1, the bands at 3485 and 3391 cm^−1^ in **H**_**3**_**PIBS** spectra were assigned to the stretching vibrations of the OH groups. NH stretching vibration band appeared at 3238 cm^−1^ [36]. The isoxazole azomethine stretching vibration peak appeared at 1596 cm^−1^, whereas the azo group had its peak appeared at 1503 cm^−1^. The two bands apparent at 1375 and 1163 cm^−1^, SO_2_ group, provided two peaks for the asymmetric and symmetrical stretching vibrations, respectively. At 1202 cm^−1^, the band corresponding to the (C-O) bond was visible.

In the spectra of metal complexes **H**_**2**_**PIBS-Cu**, **H**_**2**_**PIBS-Ni**, **H**_**2**_**PIBS-Co**, **H**_**2**_**PIBS-Fe**, and **H**_**2**_**PIBS-Zn**, the broad band appeared within the extent 3369–3456 cm−1 had assigned to the stretching vibration of OH of water molecules incorporated in the complexes structures which overlapped with the stretching vibration band of uncomplexed OH group. In the spectra of all chelates, the band appearing in the extents 4211–4240, 1361–1383, and 1162–1178 cm^−1^ had been allocated to the stretching vibration of NH, asymmetric stretching of SO_2_, and symmetrical stretching of SO_2_ group, respectively. Although these bands are not taking part in coordination with the metal centres, the shift in their positions, in some cases, refers to their involvement in the H-bong formation [37] and/or the different locative orientation of the S=O group in the complex with that of the free ligand [38]. Isoxazole ring's azomethine that appeared in the ligand spectra at 1596 cm^−1^ has appeared in the complexes' spectra in the extent of 1595–1598 cm^−1^ and thus providing a little variation in the band positions in relation to their locations in the free ligand. Such behaviour is consistent with the group nonengaging in chelation to the metal core.

Oppositely, the two bands corresponding to *v*(N=N) and (C-O) showed up in the spectra of all chelates in the extents of 1457–1480 and 1216–1222 cm^−1^, respectively, provided sufficiently large shift in their position in comparison to the band position in the free ligand, indicating their involvement in the covalent coordinate bond formation with the metal centres. The presence of a nonligand band between 433 and 568 nm that is attributed to *ν*(M-N) and (M-O) in the spectra of all metal complexes [37] provides additional evidence that nitrogen and oxygen atoms are involved in complex formation.

### 3.5. Thermogravimetric Results

**Figure 4:**
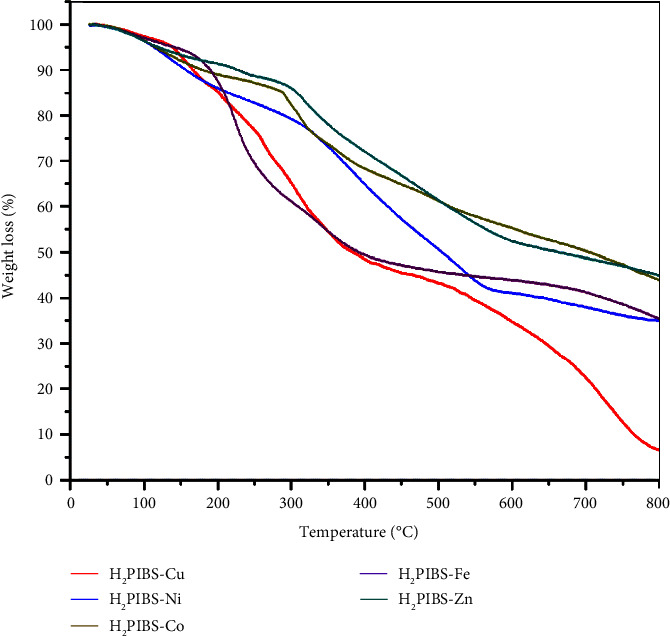
TG thermograms of metal complexes.

The thermal analysis technique, which provides crucial details on compounds' thermal properties, steps of thermal degradation, types of intermediates, and residual products, is one of the most useful approaches used to predict the molecular structure and stability of compounds. Recognizing the anionic groups linked to the metal centre as well as the quantity and kind of water and/or organic solvent molecules that attached to the metal centre is necessary. The TG thermograms of **H**_**3**_**PIBS** metal chelates are shown in Figure 4.  Table 3 also shows the analysis results of each thermogram. It is evident from the TG thermograms that the complexes under study degraded in 4 phases as shown by **H**_**2**_**PIBS-Ni** and **H**_**2**_**PIBS-Fe** complexes or 5 phases as for **H**_**2**_**PIBS-Cu**, **H**_**2**_**PIBS-Co**, and **H**_**2**_**PIBS-Zn** complexes. For **H**_**2**_**PIBS-Cu**, the first stage appeared in the range 25–105°C within which lattice water molecules are lost. The second stage successively appeared and extended to 183°C, in which coordination water was missed. For the rest of complexes, the first stage extended from 25 to 188°C, in which coordinated water molecules are lost, whereas the second step appeared in the range 113–392°C. Within this stage, coordinated chloride ions are lost alongside with a part of the organic ligand. After the second step, the decomposition of the organic ligand continued successively within two or three stages up to the end of the applied heating temperature, i.e., 800°C.

### 3.6. Geometrical Arrangements via UV-Vis Spectra and Magnetic Moment

**Figure 5:**
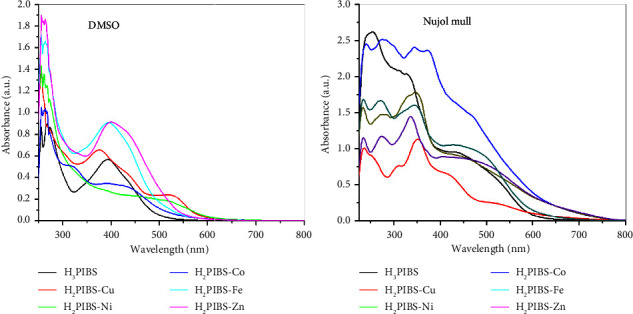
UV-Vis spectra of **H**_**3**_**PIBS** and its metal chelates in DMSO and as Nujol mull.

The produced compounds' UV-Vis spectra were obtained by dissolving the compounds in the DMSO solvent and also by following the Nujol mull process, in the 200–800 nm range; the assigned bands are collected in Table 4 and presented in Figure 5.

From the spectra of the ligand **H**_**3**_**PIBS**, the band appeared in the Nujol mull spectrum at 253 nm and in DMSO at 256 and 267 nm corresponding to the transition of the type *π *⟶ *π*∗ in the phenyl rings. The bands appearing at 275 and 395 nm in DMSO and at 328 nm in Nujol mull spectrum assigned to *π *⟶ *π*∗ and *n *⟶ *π*∗ transitions in the C=N group, respectively. The bands appearing at 435 and 523 nm in Nujol mull and at 443 nm in DMSO assigned to *n *⟶ *π*∗ transitions of N=N and SO_2_ groups.

The divalent Cu chelate, **H**_**2**_**PIBS-Cu**, exhibited the low intensity band appearing in the Nujol mull spectrum at 723 nm assigned to ^2^*E*_g_ ⟶ ^2^*T*_2g_ transition documented for octahedral Cu(II) complexes [24]. The two bands appearing at 440 and 420 nm in DMSO and Nujol mull, respectively, assigned to MLCT spectra [24]. Upon measuring the magnetic moment (*μ*_eff_), the obtained value was calculated to be 1.94 B.M characteristic for monomeric Cu(II) complexes affording no Cu-Cu interaction [39].

Ni(II) complex **H**_**2**_**PIBS-Ni**, afforded two bands in DMSO and Nujol mull at 446 and 468 nm, successively, that assigned to ^4^*A*_2g_ ⟶ ^3^*T*_1g_ (P) transition. The Nujol mull spectrum band appeared at 679 nm assigned to ^3^*A*_2g_ ⟶ ^3^*T*_1g_ (F) transition. Both these transitions are usually found in octahedral Ni(II) chelates [33, 34]. Such compound afforded the magnetic moment value (*μ*_eff_) equals to 3.32 B.M supporting high spin Ni(II) compounds.

For the Co(II) complex **H**_**2**_**PIBS-Co**, the bands appearing in the DMSO spectrum at 439 and 530 nm and in the Nujol mull spectrum at 449 and 549 nm are assigned to ^4^*T*_1g_(F) ⟶ ^4^*T*_1g_(P) and ^4^*T*_2g_(F) ⟶ ^4^A_2g_ transitions, respectively. These two transitions combined with the transition of the type ^4^*T*_1g_(F) ⟶ ^4^*T*_2g_ that corresponded to the low intensity band appearing in the Nujol mull spectrum at 692 nm are characteristic to cobalt(II) octahedral chelates [25]. Magnetic moment of 4.39 B.M has been obtained that is distinctive for high-spin octahedral Co(II) chelates.

For the trivalent Fe complex, **H**_**2**_**PIBS-Fe**, the three bands that appeared in the Nujol mull spectrum at 465, 544, and 677 nm documented to the transitions of the type ^6^*A*_1g_(S) ⟶ ^4^*A*_1g_(G), ^6^*A*_1g_(S) ⟶ ^4^*T*_2g_(G), and ^6^*A*_1g_(S) ⟶ ^4^*T*_1g_(G), respectively, afforded to d^5^ octahedral iron chelates [33, 34]. The spectrum measured in DMSO exhibited only 2 transitions at 442, 530 nm assigned to ^6^*A*_1g_(S) ⟶ ^4^*A*_1g_(G) and ^6^*A*_1g_(S) ⟶ ^4^*T*_2g_(G) transitions, successively. *μ*_eff_ value of 5.89 B.M was obtained for this high spin Fe(III) chelate.

For the Zn(II) chelate, **H**_**2**_**PIBS-Zn**, having d^10^ configuration, hence there is no available d-d electronic transition. Such compound shows just bands in visible region due to charge transfer (CT). So, the obtained spectra afforded the visible bands at 400 and 439 nm in DMSO and at 456 and 502 nm in Nujol mull that was assigned to CT transition [33, 34].

### 3.7. X-Ray Diffraction (XRD) Studies

**Figure 6:**
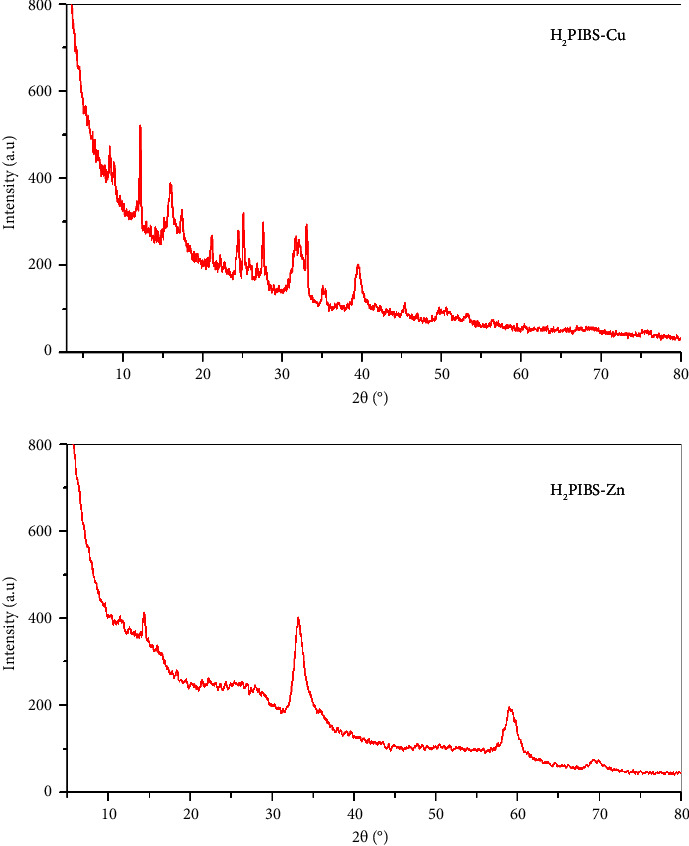
XRD spectra of **H**_**2**_**PIBS-Cu** and **H**_**2**_**PIBS-Zn** chelates.

In light of recent developments in technology and knowledge related to material science, one of the most significant techniques for providing structural microcrystalline details regarding substances being fulfilled is X-ray powder diffraction [40]. As a result, metal complexes **H**_**2**_**PIBS-Cu**, **H**_**2**_**PIBS-Ni**, **H**_**2**_**PIBS-Co**, **H**_**2**_**PIBS-Fe**, and **H**_**2**_**PIBS-Zn** that were under examination and the free ligand **H**_**3**_**PIBS** diffraction patterns were both performed within wide scattering angle range (10° < 2*θ* > 80°) and presented in Figures 6, S6, and S7. The free ligand pattern is entirely different from the pattern of the inspected metal complexes that promote complex formation and confirm that there are no smeared contaminants or initiating reactants [41]. Well-defined crystal structures were indicated by the X-ray diffraction patterns obtained for **H**_**3**_**PIBS** and complex **H**_**2**_**PIBS-Cu**, and good crystallinity was observed for complexes **H**_**2**_**PIBS-Fe** and **H**_**2**_**PIBS-Zn** with completely different diffraction patterns, whereas complexes **H**_**2**_**PIBS-Ni** and **H**_**2**_**PIBS-Co** appeared completely amorphous. Debye–Scherrer equation based on FWHM for the characteristic peak was used to determine the crystal size [42], ligand **H**_**3**_**PIBS**, complexes **H**_**2**_**PIBS-Cu**, **H**_**2**_**PIBS-Fe**, and **H**_**2**_**PIBS-Zn**, displayed average crystallite sizes 17.2, 2.4, 43.9, and 6.1 nm, respectively. The amorphous form of the complexes **H**_**2**_**PIBS-Ni** and **H**_**2**_**PIBS-Co** may be an indication of the uneven arrangement of the solid constituents during the precipitation process. If the complexation process is swift and/or the generated chemical is cooled quickly, this amorphous state will predominate. Furthermore, a crystal lattice cannot hold many of the complex's structural components. They precipitate in a phase that is primarily amorphous. The examined compounds' amorphous nature may be the reason for the aggregates' unusual small sizes, which were confirmed by TEM investigation to be in the nanometric range. The innovative nanosized obtained compounds can generate a great deal of anxiety due to their unique functional characteristics and a vast array of potential technological applications, which include enhanced catalysis, microelectronics, optics, and chemical biosensors [27].

### 3.8. TEM Images

**Figure 7:**
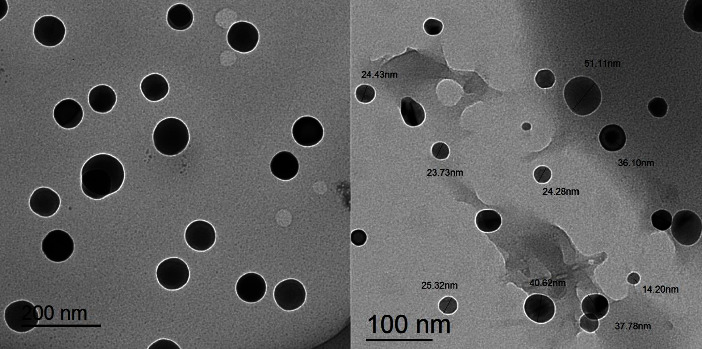
TEM images of **H**_**2**_**PIBS-Cu** chelate.

**Figure 8:**
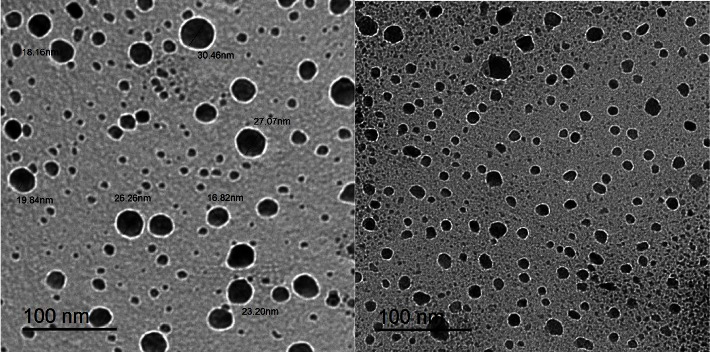
TEM images of **H**_**2**_**PIBS-Fe** chelate.

Transmittance electron microscopy (TEM) is one of the most common techniques to study a wide range of fascinating nanosized materials. It is a method that is usually employed to look at the shape and dimensions of solid materials. Therefore, the particle sizes and crystallinities of the produced complexes were further investigated by the TEM technique. Images captured by the transmittance electron microscope in bright field are shown in Figures 7, 8, and S8–S10 demonstrating that the samples are made up of tiny, different-sized nanoparticles except for complex **H**_**2**_**PIBS-Zn**.

It is obvious from these images that the metal complex powders are composed of tiny, spherical-like particles (either regular or not). In addition, it was found that the particle sizes of metal complexes ranged from 14.2 to 51.11 nm for **H**_**2**_**PIBS-Cu**, 26.47 to 29.96 nm for **H**_**2**_**PIBS-Ni**, 60.48 to 73.89 nm for **H**_**2**_**PIBS-Co**, 16.82 to 30.46 nm for **H**_**2**_**PIBS-Fe**, and 72.21 to 219.00 nm for **H**_**2**_**PIBS-Zn**.

Therefore, it is hypothesized that the biological efficiency of these novel synthetic chelates at these nanometric sizes is greater than that of their bulk analogues. The new functional characteristics of nanoscale metal complexes and their numerous potential technological applications have the potential to generate a great deal of interest [27].

### 3.9. Molecular Docking Simulation

Structure-based drug designs are an essential part of most industrial drug discovery programs. Therefore, to primarily examine the extent of the synthesized compounds' biological efficacy, their interactions with *Staphylococcus aureus* adhesion protein receptor (PDB ID: 4m01) as examples of fungal strain was studied.

**Figure 9:**
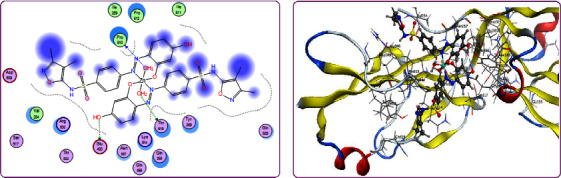
2D (a) and 3D (b) binding interactions of **H**_**2**_**PIBS-Zn** with *Staphylococcus aureus* adhesion protein.

The complete profile of the investigated substances' interactions with 4m01 is depicted in Table 5 and presented in Figures 9, as an example. Examination of the data shown in Table 5 indicates the relative strong tendency of the target compounds for docking on 4m01 which have been concluded from docking score values. These values were found to be in the range −4.9221 − −7.0350 kcal/mol with. For 4m01, the strongest binding was afforded by **H**_**2**_**PIBS-Cu** complex with docking score of −7.0350 kcal/mol. The least binding ability was observed by **H**_**2**_**PIBS-Ni** with docking score values of −4.9221 kcal/mol to 4m01. The binding ability of the tested compounds to 4m01 follow the order (form the docking score value): **H**_**2**_**PIBS-Cu** > **H**_**2**_**PIBS-Zn** > **H**_**3**_**PIBS** > **H**_**2**_**PIBS-Fe** > **H**_**2**_**PIBS-Co** > **H**_**2**_**PIBS-Ni**. The value of bond lengths for the majority of interactions that were found to be, in most cases, smaller than 3.5 Å, is the reported range for true docking track [33]. These values led to the conclusion that the investigated compounds had real docking tracks to both 4m01 and 4ynt. The collected information showed that most of the investigated compounds may have promising activity and deserve further practical examination for their biological activities, especially **H**_**2**_**PIBS-Zn**, **H**_**2**_**PIBS-Cu**, and **H**_**3**_**PIBS**.

**Figure 10:**
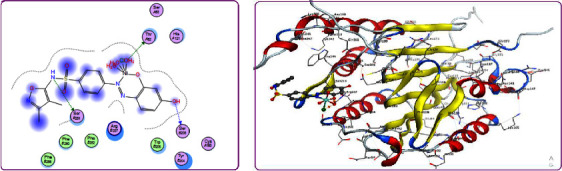
2D (a) and 3D (b) binding interactions of **H**_**2**_**PIBS-Ni** with caspase-3.

Also, to assign the extent of the antitumor activity of the tested compounds, the interaction of the compounds under study with caspase-3 (PDB ID: 2XYG) has been evaluated, Figure 10. The docking score values are found to be inside the range −5.7176 to −7.7365 kcal/mol. The strongest binding was afforded by **H**_**2**_**PIBS-Cu** complex with docking score of −7.7365 kcal/mol followed by **H**_**2**_**PIBS-Zn** complex with docking score of −7.6576 kcal/mol. The binding ability of the tested compounds to 2XYG follow the order (form the docking score value): **H**_**2**_**PIBS-Cu** > **H**_**2**_**PIBS-Zn** > **H**_**2**_**PIBS-Fe** > **H**_**2**_**PIBS-Ni** > **H**_**2**_**PIBS-Co** > **H**_**3**_**PIBS**

By docking the cocrystallized ligand of 4ynt, the docking program used in the current study has been validated. The optimal position was chosen and aligned with the docked pose of the same ligand to have the best binding energy, ligand-receptor interactions, and active site residues (Figure S11). The employed docking program performed well, with the RMSD being 1.546 [33].

### 3.10. Antibacterial and Antifungal Activities Screening

As stated in the Supplementary Material, the free ligands and complexes under consideration were tested for antibacterial and antifungal activities using the well diffusion method [29] (Table 6). Complexes **H**_**2**_**PIBS-Cu** and **H**_**2**_**PIBS-Co** showed very strong and promising antibacterial activities against *S. aureus* within inhibition zones 30 and 19 mm, which are tripple and double the applied standard amoxicillin-clavulanic inhibition zone (10 mm). Ligand **H**_**3**_**PIBS** and complexes **H**_**2**_**PIBS-Fe** and **H**_**2**_**PIBS-Zn** displayed good antibacterial activities within 9, 12, and 9 mm inhibition zones which are nearly equivalent to the standard amoxicillin-clavulanic acid (AmC 30) towards the same organism. Also, complexes **H**_**2**_**PIBS-Cu** and **H**_**2**_**PIBS-Co** displayed vigorous and favorable bactericidal influences towards *B. cereus* within inhibition nearly double (20 mm) the used standard. Ligand **H**_**3**_**PIBS** and complex **H**_**2**_**PIBS-Zn** visualized a perfect effect within 13 and 8 mm inhibition zones nearly equipollent the reference standard. Only complex **H**_**2**_**PIBS-Zn** showed excellent antibacterial efficiency against *E. coli* (16 mm) exactly double the used reference (8 nm). Furthermore, ligand **H**_**3**_**PIBS** and complex **H**_**2**_**PIBS-Co** showed strong and positive bactericidal effects towards *S. typhi* with inhibition zones (18 and 9 mm) greater than AMC30. Ligand **H**_**3**_**PIBS** and complex **H**_**2**_**PIBS-Zn** exhibited inhibition zones of 27 and 33 mm tripple the applied standard amphotericin-p (11 nm) towards *A. flavus* (multicellular fungus). Ligand **H**_**3**_**PIBS** and complexes **H**_**2**_**PIBS-Cu** and **H**_**2**_**PIBS-Co** showed fabulous and committed antifungal activities of 22, 16, and 20 mm inhibition zones against unicellular fungus (*C. albicans*) greater than amphotericin-p (13 mm). The chosen species exhibit a variety of microorganisms, such as unicellular and multicellular fungi, as well as Gram-positive and Gram-negative bacteria [43]. In most cases, the presence of metal ions, which are more hypersensitive to microbial cells than the organic chelating agent, can be blamed for the increased antimicrobial action during chelate formation [44]. The increased lipophilicity during complexation may also be the reason for the metal chelates' stronger effects. The coordinated metal ions may inhibit the activity of cellular enzymes or result in harmful interactions between cellular components. Either variations in the ribosomes in microbial cells or the impermeability of the cells of microorganisms cause obvious alterations in the activity of various complexes across different organisms [45]. According to the information at hand in the present investigation, the investigated ligands and their complexes show significant promise for developing into novel, extremely potent, broad-spectrum bactericides and fungicides.

### 3.11. Antitumor Activity Evaluation

**Figure 11:**
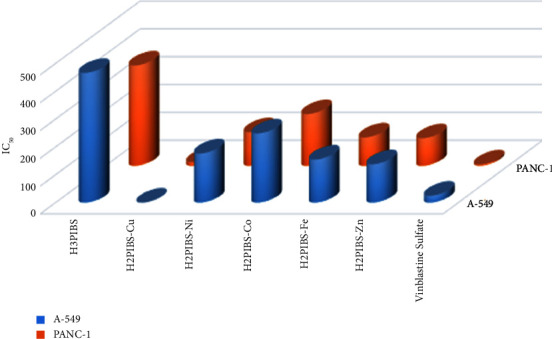
IC_50_ (*μ*g/ml) values for *in vitro* antitumor activity of **H**_**3**_**PIBS** and its metal chelates against **A-549** and **PANC-1** cancer cells.

There are currently a large number of metal-based complexes in the arsenal of cytotoxic compounds. Clinical contexts routinely make use of platinum(II) complexes, particularly those that target genomic DNA, such as cisplatin, carboplatin, and oxaliplatin. A platinum drug is given, either alone or in combination, to around 50% of cancer patients receiving chemotherapy. Platinum drugs, despite being essential in cancer chemotherapy, have substantial drawbacks, such as a systemic dose-related toxicity, limited spectrum of antitumor effectiveness, and a propensity to cause drug resistance, which usually leads to treatment failure [46]. In response to these observations, there is a lot of interest in researching nonplatinum metal-based drugs as an effective replacement [41]. In this concern, we sought to assess the new compounds' ability to inhibit the growth of human pancreatic carcinoma (Panc-1) and lung carcinoma (A-549) cell lines. The value of growth inhibitory concentration (IC_50_), which represents the concentration of the compounds needed to produce a 50% inhibition of cell growth after incubation for 72 hours, parallel to untreated controls, was used to represent the cytotoxicity results. The survival curve of the tumor cell line, which was plotted between concentration and % cell viability, was used to derive the IC_50_ values. Each test substance was diluted in a variety of quantities (1.56, 3.125, 6.25, 12.5, 25, 50, and 100 *μ*g/ml) and presented in Figure 11, and Table S1 shows the results of IC_50_ concentrations of synthetic compounds in comparison to the widely used anticancer medication vinblastine sulphate (VS) as standard which displayed IC_50_ values 24.6 and 4.68 *μ*g/ml towards lung and pancreatic carcinoma cells, respectively. Untreated cells were used as a control, and each data point was expressed as the value ± SD and represented the average of three separate studies [16]. The information acquired demonstrated that examined chemicals showed a rising propensity to colonise A-549 and Panc-1 cells lines in the following order; ligand **H**_**3**_**PIBS** ˂ **H**_**2**_**PIBS-Co** ˂ **H**_**2**_**PIBS-Ni** ˂ **H**_**2**_**PIBS-Fe** ˂ **H**_**2**_**PIBS-Zn** ˂ **H**_**2**_**PIBS-Cu**. All of the compounds under consideration decreased the viability of cancer cells, and this effect was greatly influenced by the nature of the metal ions and the geometrical arrangements surrounding the central metal ions [40]. **H**_**2**_**PIBS-Cu** complex exhibited 12.26 *μ*g/ml IC_50_ value defeating the applied standard, which proves effective and incredibly encouraging anticancer effects against A-549 cell lines. Also, **H**_**2**_**PIBS-Cu** displayed the most promising leverage towards Panc-1 cells within 13.43 *μ*g/ml IC_50_ value. These results evidenced that these compounds are very effective and auspicious for future anticancer treatments for the tumor cells being inspected.

### 3.12. Binding Mode to DNA

#### 3.12.1. UV-Vis Spectra

Among the tools which frequently applied to assign the mode of DNA binding with chemical compounds, UV-Vis titration spectra are considered a considerable one where the compound's absorption peak may alter in its position or strength as a result of its interaction with the DNA molecule [16, 47].

The spectral feature that is frequently observed in the case of intercalation binding is the absorbance decrease of the compounds' spectral peak (hypochromism) upon adding increasing amounts of DNA, which is caused by *π *⟶ *π*∗ stacking interaction. The spectral peak may also undergo red-shift (bathochromism) in case of DNA duplex stabilization. The other spectral which probably observed is the absorbance increases of the compounds' spectral peak (hyperchromism), which can be explained by the external binding of the compounds and DNA, it is sometimes accompanied by blue shift of the spectral peaks (hypsochromic shift) [24].

**Figure 12:**
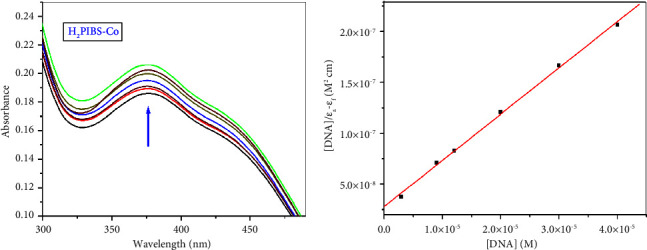
(a) Absorption spectra of constant concentrations of **H**_**2**_**PIBS-Co** with different concentrations of SS DNA. (b) Plot of [DNA] vs. [DNA]/(*εf* − *εa*).

**Figure 13:**
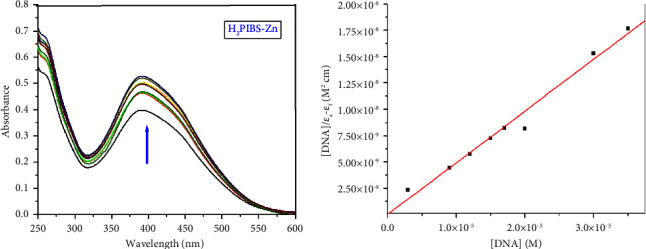
(a) Absorption spectra of constant concentrations of **H**_**2**_**PIBS-Zn** with different concentrations of SS DNA. (b) Plot of [DNA] vs. [DNA]/(*εf* − *εa*).

Measurement of UV-Vis spectra of the compounds presented in the current study have been carried out using fixed concentration of the ligand or its complexes that was taken as 50 *μ*M, and the concentration of the used SS-DNA was changed in the range of 0–40 *μ*M. The spectra were recorded for each addition of DNA. By adding an equal amount of SS-DNA to the test and reference solutions, DNA absorbance was cancelled. Following these procedures, the absorption spectra of **H**_**3**_**PIBS**, **H**_**2**_**PIBS-Cu**, **H**_**2**_**PIBS-Ni**, **H**_**2**_**PIBS-Co**, **H**_**2**_**PIBS-Fe**, and **H**_**2**_**PIBS-Zn** were measured; the spectra are illustrated in Figures 12, 13, and S12–S15 As seen in these figures, the absorption peaks illustrated at *λ* equal to 262, 369, 378, 390, and 396 nm for **H**_**3**_**PIBS, H**_**2**_**PIBS-Cu**, **H**_**2**_**PIBS-Co**, **H**_**2**_**PIBS-Fe**, and **H**_**2**_**PIBS-Zn**, successively, afforded hyperchromic behaviour. For **H**_**2**_**PIBS-Ni**, the peak appeared at 409 nm showed hypochromic behaviour. The former performance matches with the tight compounds binding to DNA, and its interactions with compounds cause DNA to stabilize, which is likely due to electrostatic contact or partial unravelling of the DNA helix structure, exposing additional DNA bases. These characteristics are signs of strong complex binding to DNA. In addition, after the complex-DNA interaction, the hyperchromic effect reflects the equivalent changes in the secondary structure of DNA in its conformation [33, 48].

The value of binding constant (*K*_*b*_), which can be obtained by application of Wolfe–Shimmer equation [48], is usually used to compare the extent of binding of the interested compounds with respect to each other and also in comparison to reported compounds such as ethidium bromide (well-known intercalator). The calculated values of *K*_*b*_ were found to be 1.74 × 10^5^ M^−1^ (**H**_**3**_**PIBS**), 5.36 × 10^5^ M^−1^ (**H**_**2**_**PIBS-Cu**), 9.52 × 10^5^ M^−1^ (**H**_**2**_**PIBS-Ni**), 1.63 × 10^5^ M^−1^ (**H**_**2**_**PIBS-Co**), 9.11 × 10^4^ M^−1^ (**H**_**2**_**PIBS-Fe**), and 1.62 × 10^6^ M^−1^ (**H**_**2**_**PIBS-Zn**) following the order (**H**_**2**_**PIBS-Zn**) > (**H**_**2**_**PIBS-Ni**) > (**H**_**2**_**PIBS-Cu**) > (**H**_**3**_**PIBS**) > (**H**_**2**_**PIBS-Co**) > (**H**_**2**_**PIBS-Fe**). The obtained values of *K*_*b*_ assured strong binding affinity of all tested compounds compared to the reported value of the most famous classical intercalator compound, EB, having *K*_*b*_ value of 1.23 × 10^5^ M^−1^ with CT DNA [24]; however, UV spectroscopic titration experiments cannot be used to solely hypothesize the precise method of binding.

#### 3.12.2. Viscosity Mensuration

Viscosity mensuration, which provides high accuracy for any change in DNA length, is unquestionably the most important hydrodynamic way of determining the binding mode between the tested drug and DNA. So, such method has received a lot of attention for identifying the kind of DNA interaction taking place in the solution.

**Figure 14:**
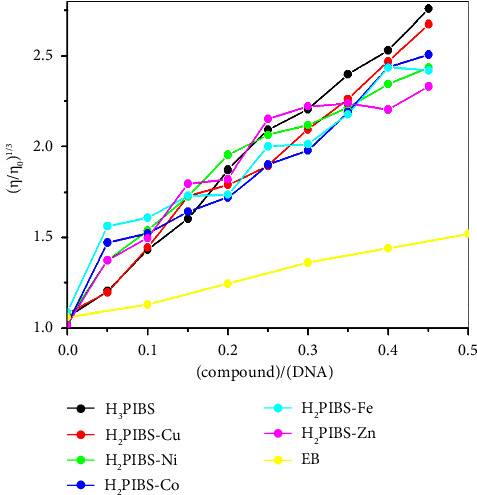
Viscosity of SS-DNA solutions containing different concentrations of **H**_**3**_**PIBS**, its complexes, and EB.

Several behaviours are probable to take place for the viscosity of DNA solution upon binding with chemical compounds. The viscosity of the DNA solution is largely increased upon increasing the concentration of chemical compounds in a behaviour familiar to the classical intercalation mode of binding for such binding to occur, a big distance between the base pairs is needed leading to an enhancement of the DNA solution viscosity [49]. This behaviour is a common behaviour for classical intercalator such as EB [50]. Another typical behaviour is the slight rise/decrease or even no change in DNA viscosity with raising the compounds' concentration that may be due to bending or kinking in the DNA double helix. This behaviour reduces the effective DNA length and supports nonclassical and/or partial intercalation interaction in the DNA grooves [51, 52]. So, in order to get full insight about the exact mode of binding of the compounds under interest and the SS-DNA, solutions containing 3 × 10^−5^ M of DNA and increasing concentrations of the investigated compounds (1.5 × 10^−6^ to 1.5 × 10^−5^ M) were prepared, and their viscosity were measured in addition to the blank solution (containing only 3 × 10^−5^ M of DNA). The results are shown in Figure 14 along with the reported data of EB. As shown in this figure, all the tested compounds afforded large increase in the DNA viscosity upon gradually increasing the investigated compounds' concentrations, the viscosity of all compounds exceeds that of EB demonstrating that the predominant way of binding for all compounds with SS-DNA is intercalative.

## 4. Conclusion

**Scheme 1:**
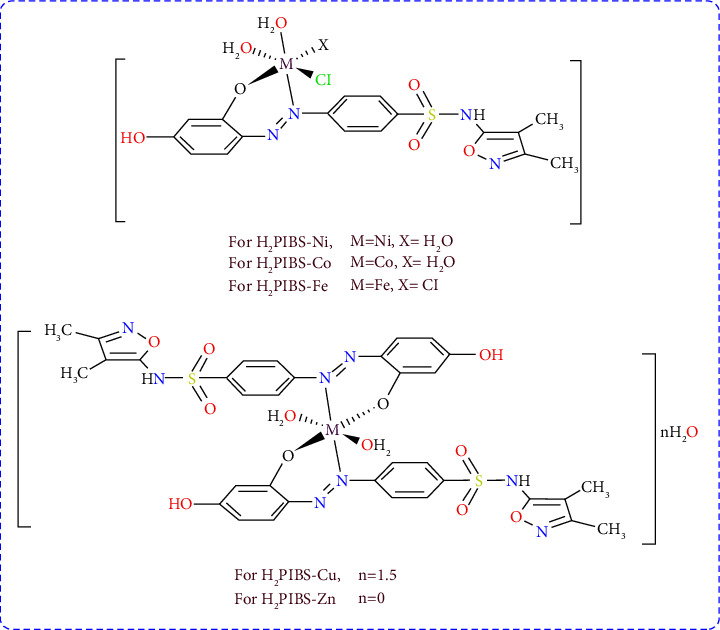
Chem draw complexes structures.

Cu(II), Ni(II), Co(II), Fe(III), and Zn(II) complexes of the azo dye formed by the coupling of sulfafurazole diazonium chloride with resorcinol have been isolated. Structure investigation of the isolated chelates have been obtained through spectroscopic and analytical tools, which collaborated to assure the formation of the metal chelates in the molecular formulae, [Cu(H_2_PIBS)_2_(H_2_O)_2_]·1.5H_2_O, [Ni(H_2_PIBS)Cl(H_2_O)_3_], [Co(H_2_PIBS)Cl(H_2_O)_3_], [Fe(H_2_PIBS)Cl_2_(H_2_O)_2_], and [Zn(H_2_PIBS)_2_(H_2_O)_2_], where H_2_PIBS abbreviates the monodeprotonated ligand, and hence indicating the formation of 1L: 1M stoichiometry for Ni(II), Co(II), and Fe(III) chelates, where Cu(II) and Zn(II) complexes formed in the ratio 2L : 1M. The geometrical arrangement around the metal canters was concluded from UV-Vis spectra to be octahedral for all metal chelates. The linkage of ligand to the metal centre occurred through the azo group nitrogen and o-hydroxyl oxygen through proton displacement, and thus the structures of the metal chelates are formulated in Scheme 1. The antimicrobial activity of the compounds under interest has been evaluated against alternative microorganisms covering the Gram positive, Gram negative bacteria, and fungi classes of microorganisms. The obtained results showed that the ligand **H**_**3**_**PIBS** and its complexes **H**_**2**_**PIBS-Cu** and **H**_**2**_**PIBS-Zn** afforded the highest activity against the alternative tested strains. Such data are in good accordance with the molecular docking simulation of the interested compounds with the receptor of *Staphylococcus aureus* adhesion protein (PDB ID: 4m01). The antitumor activity of the compounds under study has been evaluated against the tumor cell lines A-549 (human lung carcinoma cancer cell line) and Panc-1 (pancreatic carcinoma), and the results obtained showed that enhanced antitumor activity for the metal complexes in comparison with the parent ligand. Promising activity has been observed for the Cu(II) chelate with IC_50_ of 12.26 and 13.43 *μ*g/ml against A-549 and Panc-1 cell lines, respectively, that is in accordance with docking studies with caspase-3 (PDB ID: 2XYG). The mode of interaction of the compounds DNA has been examined by UV-Vis spectra and viscosity studies indicating the intercalation mode of binding with SS-DNA, which has been concluded from the *K*_*b*_ values and the enhancement of DNA solution viscosity upon adding different concentrations of the compounds under interest.

## Figures and Tables

**Table 1:** The molecular weights, microanalysis, colours, molecular formulae, empirical formulae, and molar conductance data of **H**_**3**_**PIBS** and its metal chelates.

No	Molecular formula (empirical formulae)	M. Wt. M.p. (°C)	Colour (Λ_*m*_)^*∗*^	Microanalysis found (calc.) (%)			
				%C	%H	%N	%M
**H**_**3**_ **PIBS**	(C_17_H_16_N_4_O_5_S)	388.40	Reddish orange (−)	52.74 (52.57)	4.29 (4.15)	14.56 (14.43)	—
		180					

**H**_**2**_ **PIBS-Cu**	[Cu(H_2_PIBS)_2_(H_2_O)_2_]·1.5H_2_O (C_34_H_37_CuN_8_O_13.5_S_2_)	901.38	Dark brown (25.11)	45.41 (45.30)	4.21 (4.14)	12.22 (12.43)	7.23 (7.05)
		>300					

**H**_**2**_ **PIBS-Ni**	[Ni(H_2_PIBS)Cl(H_2_O)_3_] (C_17_H_21_ClNiN_4_O_8_S)	535.58	Dark brown (29.62)	38.24 (38.12)	3.69 (3.95)	10.28 (10.46)	11.25 (10.96)
		>300					

**H**_**2**_ **PIBS-Co**	[Co(H_2_PIBS)Cl(H_2_O)_3_] (C_17_H_21_ClCoN_4_O_8_S)	535.82	Dark brown (25.77)	38.23 (38.11)	3.86 (3.95)	10.25 (10.46)	11.17 (11.00)
		>300					

**H**_**2**_ **PIBS-Fe**	[Fe(H_2_PIBS)Cl_2_(H_2_O)_2_] (C_17_H_19_Cl_2_FeN_4_O_7_S)	550.17	Dark brown (33.92)	37.32 (37.11)	3.54 (3.48)	10.28 (10.18)	10.31 (10.15)
		>300					

**H**_**2**_ **PIBS-Zn**	[Zn(H_2_PIBS)_2_(H_2_O)_2_] (C_34_H_34_ZnN_8_O_12_S_2_)	876.20	Dark brown (28.11)	46.27 (46.61)	3.78 (3.91)	12.73 (12.79)	7.78 (7.46)
		>300					

^*∗*^Λ_*m*_ is the molar conductance (Ω^−1^·cm^2^·mol^−1^).

**Table 2:** Assignments of important vibrational spectral bands of **H**_**3**_**PIBS** and its metal chelates.

Comp	*ν* (OH)	*ν* (NH)	*ν* (C=N)_ring_	*ν* (N=N)	*ν* (SO_2_)	*ν* (C-O)	*ν* (M-O)	*ν* (M-N)
**H**_**3**_ **PIBS**	3485	3238	1596	1503	1375, 1163	1202	—	—
	3391							

**H**_**2**_ **PIBS-Cu**	3456	3225	1595	1467	1361, 1162	1222	568	469

**H**_**2**_ **PIBS-Ni**	3420	3240	1598	1472	1378, 1167	1220	501	485

**H**_**2**_ **PIBS-Co**	4511	3211	1596	1457	1375, 1164	1220	521	433

**H**_**2**_ **PIBS-Fe**	3369	3217	1595	1474	1378, 1165	1216	555	467

**H**_**2**_ **PIBS-Zn**	3402	3230	1598	1480	1383, 1178	1222	527	476

**Table 3:** Thermal decomposition results of metal chelates.

No. empirical formula (mol. Wt.)	Temp. range (°C)	Mass loss (%)		Assignment
		Calc.	Found	
**H**_**2**_ **PIBS-Cu [Cu(H**_**2**_ **PIBS)**_**2**_ **(H**_**2**_ **O)**_**2**_ **]·1.5H**_**2**_ **O 901.38**	25–105	2.99	3.01	(i) Loss of 1.5 lattice H_2_O
	105–183	9.43	9.52	(ii) Loss of 2 coordinated H_2_O + 0.5O_2_ + 0.5H_2_ + CH_3_
	183–276	17.76	17.52	(iii) Loss of C_4_H_4_N_2_O_3_S fraction
	276–391	20.77	20.84	(iv) Loss of C_6_H_4_ + C_5_H_7_N_2_O fractions
	391–800	41.20	41.40	(v) Loss of C_17_H_15_N_4_O_4_S fraction

**H**_**2**_ **PIBS-Ni [Ni(H**_**2**_ **PIBS)Cl(H**_**2**_ **O)**_**3**_ **] 535.58**	25–188	13.26	12.97	(i) Loss of 3 coordinated H_2_O + 0.5O_2_ + 0.5H_2_ +
	188–392	20.82	21.03	(ii) Loss of 0.5Cl_2_ + C_6_H_4_ fraction
	392–571	23.17	23.36	(iii) Loss of C_3_H_6_NO fraction + N_2_

**H**_**2**_ **PIBS-Co [Co(H**_**2**_ **PIBS)Cl(H**_**2**_ **O)**_**3**_ **] 535.82**	25–174	10.07	9.91	(i) Loss of 3 coordinated H_2_O
	174–322	12.59	12.85	(ii) Loss of 0.5Cl_2_ + CH_3_
	322–389	7.66	7.86	(iii) Loss of C_2_H_3_N fraction
	389–577	10.27	10.61	(iv) Loss of C_2_H_2_NO fraction

**H**_**2**_ **PIBS-Fe [Fe(H**_**2**_ **PIBS)Cl**_**2**_ **(H**_**2**_ **O)**_**2**_ **] 550.17**	25–148	6.54	5.94	(i) Loss of 2 coordinated H_2_O
	148–273	30.35	29.72	(ii) Loss of Cl_2_ + C_5_H_6_NO fraction
	273–454	17.46	17.72	(iii) Loss of 0.5O_2_ + 0.5H_2_ + SO_2_NH fraction

**H**_**2**_ **PIBS-Zn [Zn(H**_**2**_ **PIBS)**_**2**_ **(H**_**2**_ **O)**_**2**_ **] 876.20**	25–113	4.10	4.42	(i) Loss of 2 coordinated H_2_O
	113–249	6.86	6.89	(ii) Loss of 4CH_3_ groups
	249–368	13.35	13.46	(iii) Loss of 0.5O_2_ + 0.5H_2_ + C_3_H_3_N_2_O fraction
	368–616	24.10	23.53	(iv) Loss of SO_2_ + C_3_H_3_N_2_O_3_S fractions

**Table 4:** Electronic absorption spectral results and *μ*_eff_ values of the metal complexes.

Compound	Wavelength (nm) in DMSO	Wavelength (nm) using Nujol mull technique	*μ *_eff_ (B.M.)
**H**_**3**_ **PIBS**	256, 267, 275, 395, 443	253, 328, 435	—
**H**_**2**_ **PIBS-Cu**	378, 440, 524	254, 308, 355, 420, 524, 723	1.94
**H**_**2**_ **PIBS-Ni**	383, 446, 517	278, 341, 373, 468, 679	3.32
**H**_**2**_ **PIBS-Co**	318, 395, 439, 530	269, 329, 351, 449, 549, 692	4.39
**H**_**2**_ **PIBS-Fe**	337, 395, 442, 530	275, 335, 465, 544 677	5.89
**H**_**2**_ **PIBS-Zn**	275, 400, 439	272, 310, 322, 347, 390, 456, 502	Diamagnetic

**Table 5:** Molecular interactions of **H**_**3**_**PIBS** and its complexes on *Staphylococcus aureus* adhesion protein (PDB ID: 4m01) and caspase-3 (PDB ID: 2XYG).

Compound	Protein PDB	Ligand moiety	Receptor site	Type of interaction	Distance (Å)	E (kcal/mol)	Docking score (kcal/mol)
**H**_**3**_ **PIBS**	4m01	O17	N THR 515 (A)	H-acceptor	3.02	−3.2	−5.6744
	2XYG	N1	NE ARG 207 (B)	H-acceptor	2.94	−3.1	−5.8634
		5-ring	NH2 ARG 207 (B)	Pi-cation	4.17	−1.5	

**H**_**2**_ **PIBS-Cu**	4m01	N14	OD1 ASN 257 (A)	H-donor	3.27	−3.9	−7.0350
		O18	N THR 258 (A)	H-acceptor	3.03	−3.2	
		O61	N THR 515 (A)	H-acceptor	3.11	−2.7	
		5-ring	CB THR 515 (A)	Pi-H	4.43	−0.6	
	2XYG	O17	SG CYS 163 (A)	H-acceptor	3.47	−0.7	−7.7365

**H**_**2**_ **PIBS-Ni**	4m01	O47	O PRO 513 (A)	H-donor	3.06	−3.0	−4.9221
		O47	OG1 THR 515 (A)	H-donor	2.86	−9.0	
		O48	O PRO 513 (A)	H-donor	3.01	−8.1	
		N31	NE2 GLN 268 (A)	H-acceptor	3.08	−0.8	
	2XYG	N14	OG SER 251 (B)	H-donor	3.04	−2.6	−6.0285
		O41	O SER 205 (B)	H-donor	3.01	−4.2	
		O46	OG1 THR 62 (A)	H-donor	3.14	−3.9	

**H**_**2**_ **PIBS-Co**	4m01	O48	O PRO 513 (A)	H-donor	3.13	−1.7	−5.4316
		O48	OG1 THR 515 (A)	H-donor	3.17	−0.5	
		6-ring	N THR 515 (A)	Pi-H	4.60	−0.8	
	2XYG	N 14	O PHE 250 (B)	H-donor	3.59	−0.9	−5.7176
		CL45	O SER 63 (A)	H-donor	3.64	−0.9	
		N1	NE1 TRP 214 (B)	H-acceptor	3.38	−1.1	

**H**_**2**_ **PIBS-Fe**	4m01	O17	NZ LYS 514 (A)	H-acceptor	3.44	−2.4	−5.6017
		O17	N THR 515 (A)	H-acceptor	3.36	−1.4	
	2XYG	6-ring	N PHE 250 (B)	Pi-H	4.22	−0.6	−6.0783

**H**_**2**_ **PIBS-Zn**	4m01	O83	OE2 GLU 493 (A)	H-donor	2.74	−8.3	−6.5594
		O 86	O PRO 513 (A)	H-donor	2.87	−9.7	
		O87	OG1 THR 515 (A)	H-donor	3.03	−5.0	
	2XYG	O18	N GLY 122 (A)	H-acceptor	3.50	−0.9	−7.6576

**Table 6:** Antibacterial and antifungal activities of **H**_**3**_**PIBS** and its metal chelates as inhibition zone diameter (mm) at 30°C after 24 h.

Complex	*S. aureus*	*B. cereus*	*E. coli*	*S. typhi*	*A. flavus*	*C. albicans*
**H**_**3**_ **PIBS**	9	13	—	18	27	22
**H**_**2**_ **PIBS-Cu**	30	20	—	—	—	16
**H**_**2**_ **PIBS-Ni**	—	—	—	—	—	—
**H**_**2**_ **PIBS-Co**	19	20	—	9	—	20
**H**_**2**_ **PIBS-Fe**	12	—	—	—	—	—
**H**_**2**_ **PIBS-Zn**	9	8	16	—	33	—
**Amoxycillin-clavulinic acid (AmC 30)**	10	12	8	8	—	—
**Amphotericin-p**	—	—	—	—	11	13

## Data Availability

The data that support the findings of this study are available from the corresponding authors upon reasonable request.

## Supplementary Materials

Supplementary Materials Section S1: instruments and methods; Section S2: antibacterial and antifungal screening; Section S3: cytotoxicity evaluation using viability assay; Section S4: molecular docking studies; Section S5: DNA-binding studies; Figure S1: ^1^H-NMR spectra of H_3_PIBS in d^6^-DMSO/D_2_O; Figure S2–S6: mass spectrum of H_3_PIBS, H_2_PIBS-Cu, H_2_PIBS-Ni, and H_2_PIBS-Fe; Figure S6, S7: XRD spectrum of H_3_PIBS and XRD and spectrum of H_3_PIBS-Fe; Figure S8–10: TEM image of H_2_PIBS-Ni, H_2_PIBS-Co, and H_2_PIBS-Zn; Figure S11: superimposition of the 4ynt cocrystallized ligand and the docked pose of the same ligand, RMSD = 1.546 Å; Figure S12–15: absorption spectra of constant concentrations of H_3_PIBS, H_2_PIBS-Cu, H_2_PIBS-Ni, and H_2_PIBS-Fe with different concentrations of SS DNA. Along with the plot of (DNA) vs. (DNA)/(*εf* − *εa*); Table S1: the *in vitro* antitumor activity (IC_50_) of the H_3_PIBS and its complexes against A-549 and PANC-1 cell line (Supplementary Materials file).
