# Supplementary material for: Metal Chelates of Sulfafurazole Azo Dye Derivative: Synthesis, Structure Affirmation, Antimicrobial, Antitumor, DNA Binding, and Molecular Docking Simulation
Source: Bioinorg Chem Appl. 2023 Apr 22;2023:2239976. doi: 10.1155/2023/2239976 (PMC10234726; doi:10.1155/2023/2239976)
Supplement: Supplementary Materials — Section S1: instruments and methods; Section S2: antibacterial and antifungal screening; Section S3: cytotoxicity evaluation using viability assay; Section S4: molecular docking studies; Section S5: DNA-binding studies; Figure S1: 1H-NMR spectra of H3PIBS in d6-DMSO/D2O; Figure S2–S6: mass spectrum of H3PIBS, H2PIBS-Cu, H2PIBS-Ni, and H2PIBS-Fe; Figure S6, S7: XRD spectrum of H3PIBS and XRD and spectrum of H3PIBS-Fe; Figure S8–10: TEM image of H2PIBS-Ni, H2PIBS-Co, and H2PIBS-Zn; Figure S11: superimposition of the 4ynt cocrystallized ligand and the docked pose of the same ligand, RMSD = 1.546 Å; Figure S12–15: absorption spectra of constant concentrations of H3PIBS, H2PIBS-Cu, H2PIBS-Ni, and H2PIBS-Fe with different concentrations of SS DNA. Along with the plot of (DNA) vs. (DNA)/(εf − εa); Table S1: the in vitro antitumor activity (IC50) of the H3PIBS and its complexes against A-549 and PANC-1 cell line (Supplementary Materials file). [file 2239976.f1.docx]

**Metal Chelates of Sulfafurazole Azo dye derivative: Synthesis, Structure Affirmation, Antimicrobial, Antitumor, DNA binding and Molecular Docking Simulation**

**Hoda A. El-Ghamry ^1,2^*, Rajaa O. Al-Ziyadi ^1^*, Fatmah M. Alkhatib^1^, Khadiga M Takroni^1^, Abdalla M. Khedr ^2^**

^1^ Chemistry Department, Faculty of Applied Science, Umm Al-Qura University, Makkah, Saudi Arabia

^2^ Chemistry Department, Faculty of Science, Tanta University, Tanta, Egypt

* Corresponding authors emails: [haelghamry@uqu.edu.sa](mailto:haelghamry@uqu.edu.sa), [helghamrymo@yahoo.com](mailto:helghamrymo@yahoo.com) (H.A. El-Ghamty); s44285650@st.uqu.edu.sa (R.O. Al-Ziyadi)

**Section S1. Instruments and methods**

Melting points were measured on a Gallenkamp melting point apparatus without corrections. The infrared spectra were recorded on a Perkin-Elmer FTIR 1430 spectrophotometer using the KBr disk. Shimadzu spectrometer (model UV-3600) was used to measure the UV-Vis spectra. Magnetic susceptibility of the paramagnetic solid chelates was recorded at ordinary temperature using magnetic susceptibility instrument applying the Gouy's technique using Hg[Co(SCN)_4_] as a calibrant. Thermogravimetric analysis (TGA) of the novel solid complexes were measured using TG-50-Schimadzu thermogravimetric analyzer under 10 ^°^C/min heating rate and N_2_ atmosphere within temperature ranging from 25-800 ^°^C. Mass spectra were measured on a Finnigan MAT 8222 EX mass spectrometer at 70 eV. Element analysis (C, H, N) was also carried out at Regional Centre for Mycology and Biotechnology (RCMB), Al-Azhar University, and the values were found to be within ± 0.4% of the theoretical ones unless otherwise indicated. Metal content was determined using inductive coupled plasma (PerkinElmer/Optima 7000 DV) after complete decomposition of the complexes in concentrated HNO_3_ several times. The X-ray diffraction (XRD) of the investigated compounds were performed using, Bruker advanced D8 Kristalloflex (Ni-filtered Cu Kα1 radiation; 1.5406 Å) for inspecting their crystal structure. The transmission electron microscopy (TEM) images were taken at 200 kV using HRTEM (JEM 2100) (JEOL, Tokyo) instrument where the samples were loaded on carbon coated Cu grids (200 mesh) and examined.

**Section S2. Antibacterial and antifungal screening**

*Preparation of bacterial and fungal inoculum*

[*Escherichia Coli*](https://www.usmslab.com/e-coli/)*,* [*Salmonella*](https://www.who.int/news-room/fact-sheets/detail/salmonella-(non-typhoidal)) typhi*,* [*Staphylococcus Aureus*](https://www.ncbi.nlm.nih.gov/books/NBK441868/)*, Bacillus cereus, Aspergillus flavus* and *Candida albicans* were sub-cultured from stock cultures in sterile bottles containing Nutrient broth incubated over-night at 37°C. Directly, prior to the experiment, fresh microbial cultures were adjusted to 0.5 McFarland to be equivalent to about 10^6^ CFU/ml.

*Antimicrobial screening applying well diffusion method*

In the antimicrobial screening, each of the compounds was dissolved in DMSO, and solution of the concentration 1 mg/mL was prepared separately; Antimicrobial activities of compounds were measured using the well agar diffusion method [1]. Each bacterial suspension (10^6^ CFU/mL) was spread on the surface of MHA plates. 10 μL of each compound were loaded on the wells in agar place. Standard disk of Amoxycillin-clavulanic acid (AmC-30) (antibacterial agent) and Amphotericin-p (Antifungal agent) served as control positive. The plates were incubated at 37 ^o^C for 12-24 hours. Evidence of clear zone indicates bacterial growth inhibition and was measured in mm. All tests were performed in triplicate

**Section S3. Cytotoxicity evaluation using viability assay:**

The applied cell lines **A-549** cells (human Lung cancer cell line) and **Panc-1** (Pancreatic carcinoma) were obtained from the American Type Culture Collection (ATCC, Rockville, MD). The cells were propagated in Dulbecco’s modified Eagle’s medium (DMEM) supplemented with 10% heat-inactivated fetal bovine serum, 1% L-glutamine, HEPES buffer and 50µg/ml gentamycin. All cells were maintained at 37ºC in a humidified atmosphere with 5% CO_2_ and were subcultured two times a week. For cytotoxicity assay, the cells were seeded in 96-well plate at a cell concentration of 1×10^4^ cells per well in 100µl of growth medium. Fresh medium containing different concentrations of the test sample was added after 24 h of seeding. Serial two-fold dilutions of the tested chemical compound were added to confluent cell monolayers dispensed into 96-well, flat-bottomed microtiter plates (Falcon, NJ, USA) using a multichannel pipette. The microtiter plates were incubated at 37ºC in a humidified incubator with 5% CO_2_ for a period of 24 h. Three wells were used for each concentration of the test sample. Control cells were incubated without test sample and with or without DMSO. The little percentage of DMSO present in the wells (maximal 0.1%) was found not to affect the experiment. After incubation of the cells for at 37°C, for 24 h, the viable cells yield was determined by a colorimetric method.

In brief, after the end of the incubation period, media were aspirated, and the crystal violet solution (1%) was added to each well for at least 30 minutes. The stain was removed, and the plates were rinsed using tap water until all excess stain is removed. Glacial acetic acid (30%) was then added to all wells and mixed thoroughly, and then the absorbance of the plates were measured after gently shaken on Microplate reader (TECAN, Inc.), using a test wavelength of 490 nm. All results were corrected for background absorbance detected in wells without added stain. Treated samples were compared with the cell control in the absence of the tested compounds. All experiments were carried out in triplicate. The cell cytotoxic effect of each tested compound was calculated**.** The optical density was measured with the microplate reader (SunRise, TECAN, Inc, USA) to determine the number of viable cells and the percentage of viability was calculated as [(ODt/ODc)]x100% where ODt is the mean optical density of wells treated with the tested sample and ODc is the mean optical density of untreated cells. The relation between surviving cells and drug concentration is plotted to get the survival curve of each tumor cell line after treatment with the specified compound. The 50% inhibitory concentration (IC_50_), the concentration required to cause toxic effects in 50% of intact cells, was estimated from graphic plots of the dose response curve for each conc. using Graphpad Prism software (San Diego, CA. USA).

**Section S4. Molecular docking studies**

The used software is MOE-Dock 2014 software. Chemical structures of the investigated compounds have been drawn using the builder of the MOE and minimization of energy has been achieved using the program force field MMFF94x. Preparation of protein including addition of H-atoms and the removal of the undesired H_2_O and other molecules has been performed prior to the docking process. Finally, docking of the 3D conformers was performed, using rescoring 1(London dG) and rescoring 2 (GBVI/WSA dG). “Ligand Interactions” tool was applied for the visualizing of the 2D protein-compound interactions illustrating all the available interactions.

**Section S5. DNA binding studies**

SS-DNA (salmon sperm DNA) was used in studying its binding mode with some selected compounds. All the experiments in this section were carried out in tris hydrochloride buffering medium (Tris–HCl buffer solution is prepared by mixing 50 mM of NaCl and 5.0 mM of tris (hydroxymethyl)-aminomethane) then adjusting the pH to be 7.2 using HCl solution).

Absorption titration experiments have been implemented while fixing the concentrations of the tested compounds and gradually raising the DNA concentration. An equal DNA concentration has been supplied to both the compound and reference solutions to abstract the DNA absorbance.

Quantitative comparison of the DNA-binding ability of the compounds under study can be performed from the calculation of their intrinsic binding constants K_b_ to DNA which can be obtained from monitoring the changes in the absorption bands according to the following equation:

[DNA]/(ε_a_ - ε_f_) = [DNA]/(ε_b_ - ε_f_) + 1/ [K_b_(ε_b_ - ε_f_)] Eq (1)

Where [DNA] is the DNA concentration solution in the base pairs. The absorption coefficients ε_a_ equals A_obs_/[compound] while ε_f_ and ε_b_ refers to the extinction coefficient of the unbounded and the compound in a fully bound state to DNA, respectively. The plot of [DNA]/(ε_a_–ε_f_) versus [DNA] is a straight line with slope = 1/(ε_b_–ε_f_) and intercept = 1/K_b_ (ε_b_–ε_f_); K_b_ is calculated from the ratio of slope to intercept.

Alternatively, viscosities of constant concentrations of SS-DNA solutions were recorded while gradually increasing the concentration of the compounds under interest. Viscosity measurements were performed using Ubbelodhe viscometer at 25 ± 0.1 ^°^C. Three replicates mean flow times were recorded for each mixture. The obtained data were interpreted as (η/η_0_)^1/3^ vs. the fraction [compound]/[DNA], where η and η_0_ are the viscosity of DNA in existence and lake of the checked compound, successively.


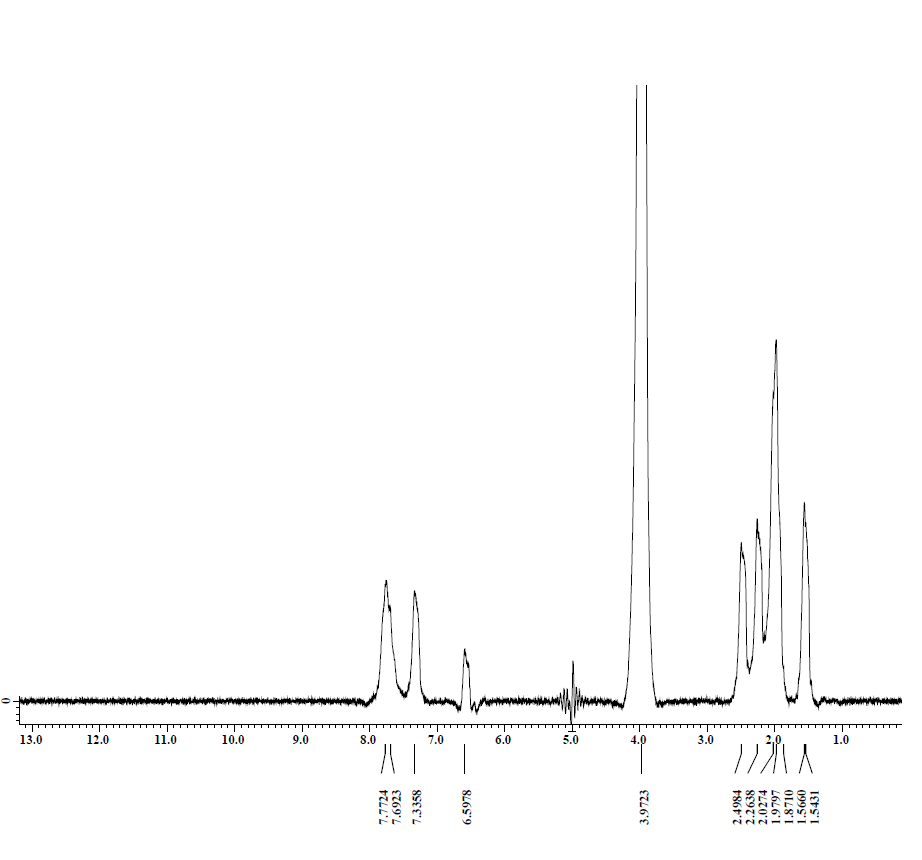


**Figure S1.** ^1^H NMR spectra of **H_3_PIBS** in d^6^-DMSO/D_2_O



**FIGURE S2** Mass spectrum of **H_3_PIBS**



**FIGURE S3** Mass spectrum of **H_2_PIBS-Cu**





**FIGURE S4** Mass spectrum of **H_2_PIBS-Ni**

**FIGURE S5** Mass spectrum of **H_2_PIBS-Fe**



**FIGURE S6** XRD spectrum of **H_3_PIBS** ligand
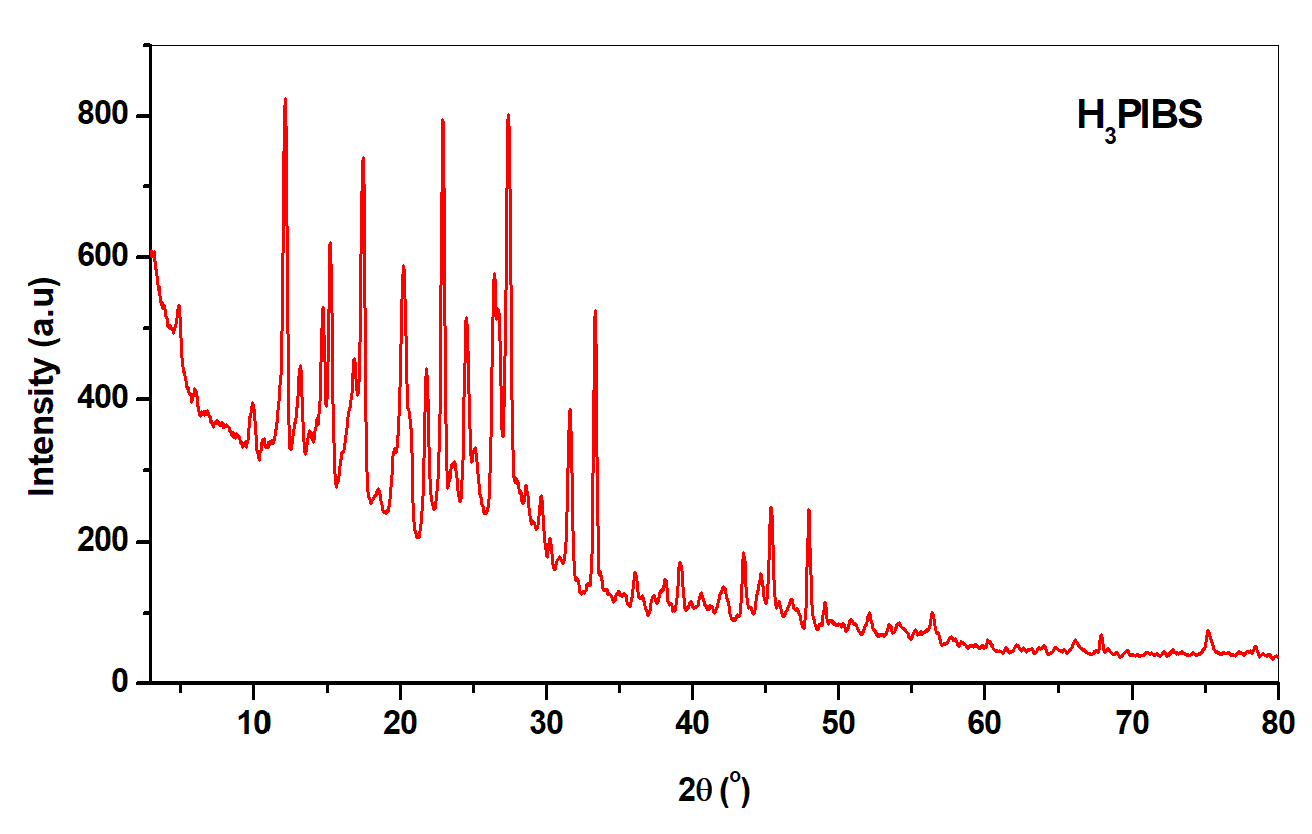


**FIGURE S**
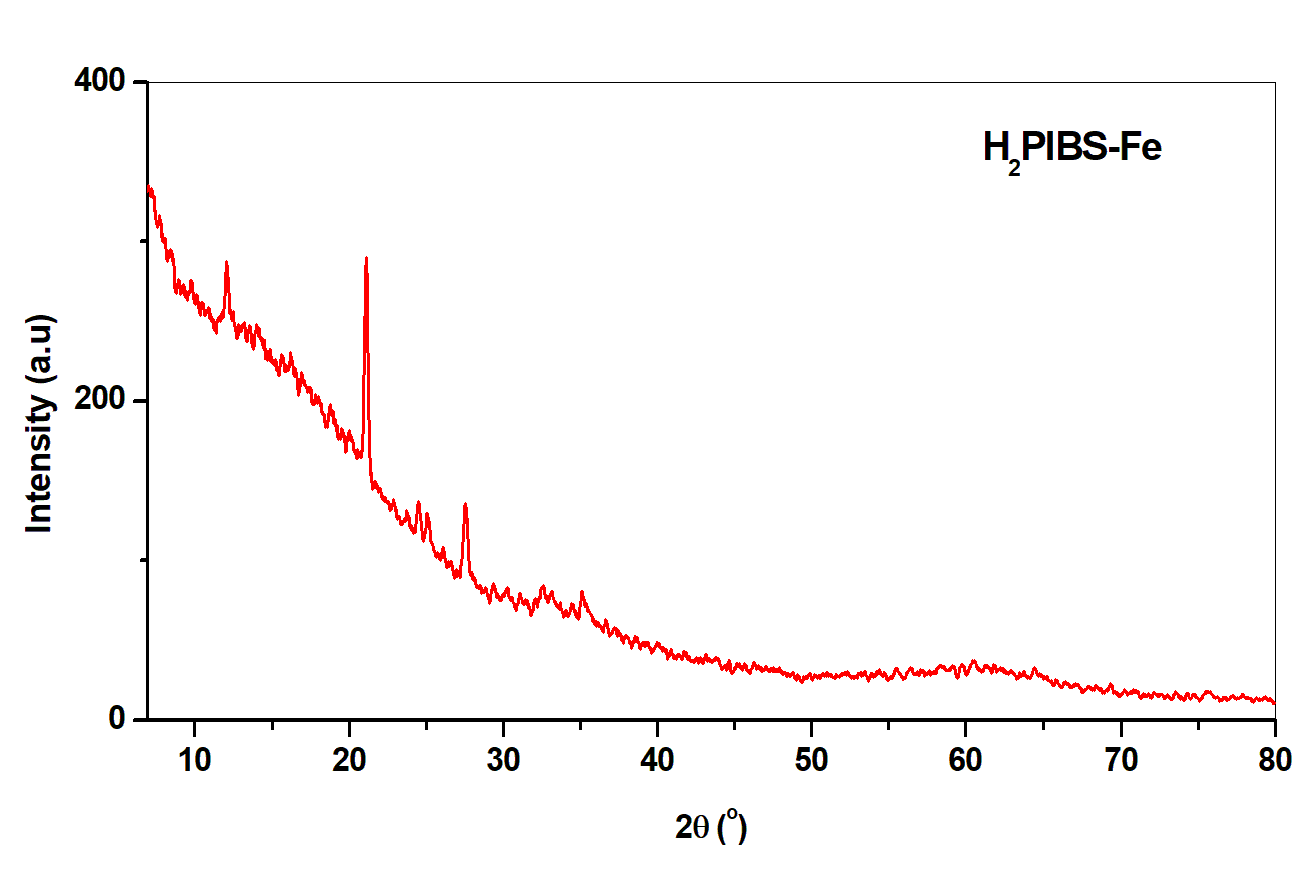
**7** XRD spectrum of **H_2_PIBS-Fe** chelate


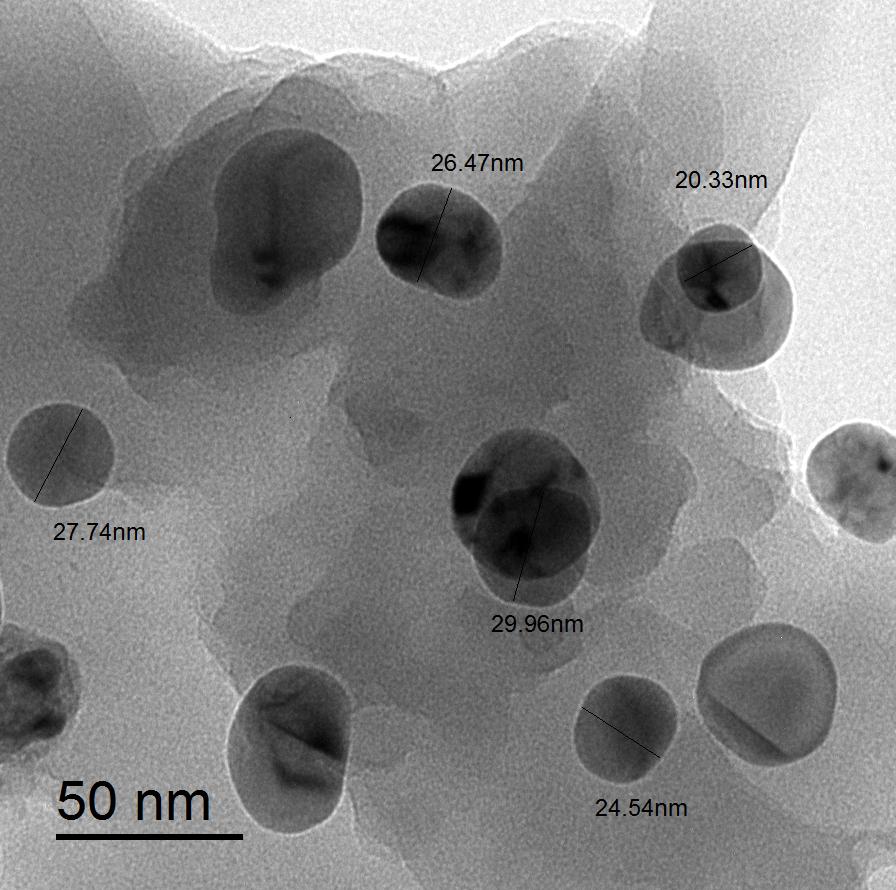


**FIGURE S8** TEM image of **H_2_PIBS-Ni** chelate


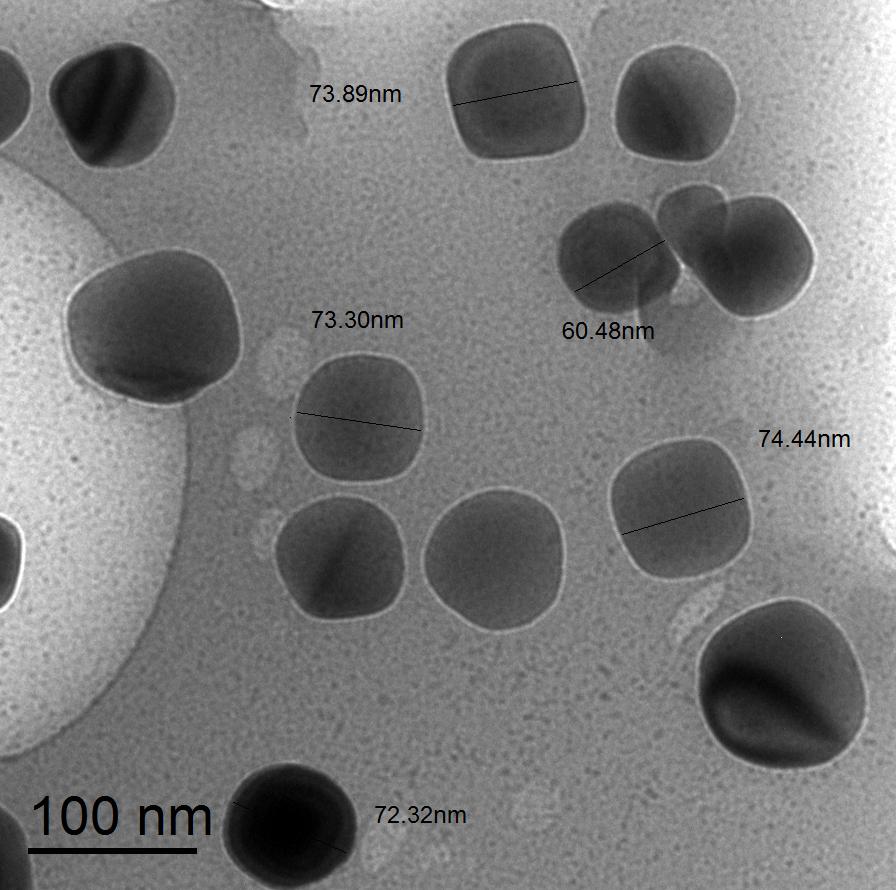


**FIGURE S9** TEM image of **H_2_PIBS-Co** chelate


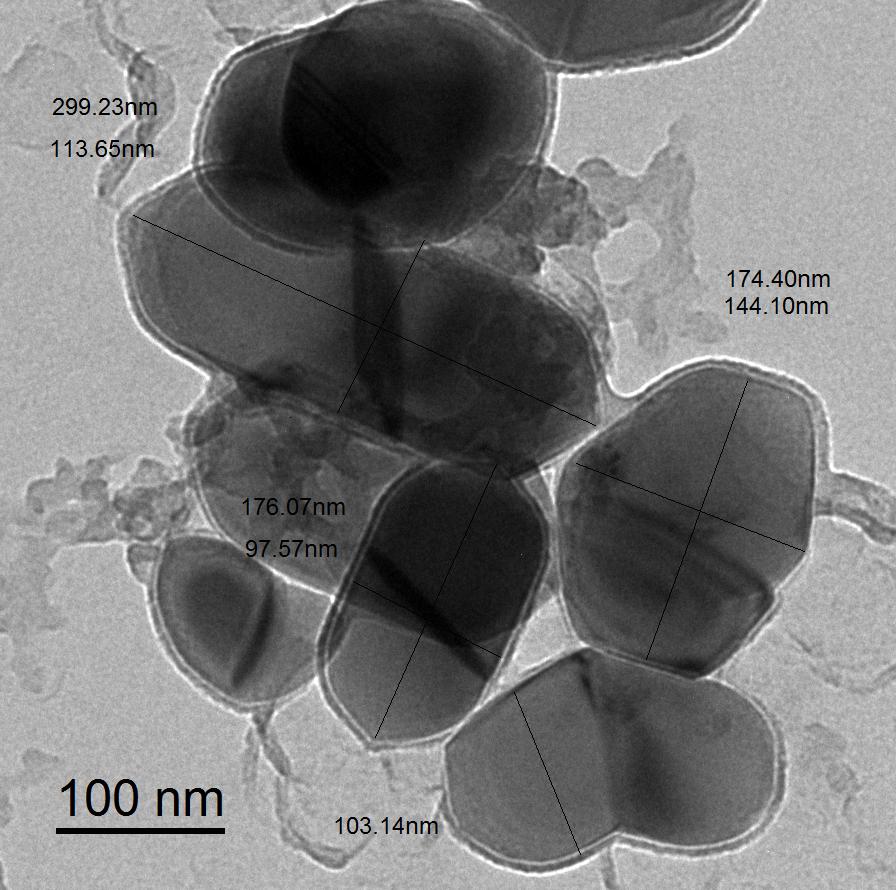


**FIGURE S10** TEM image of **H_2_PIBS-Zn** chelate


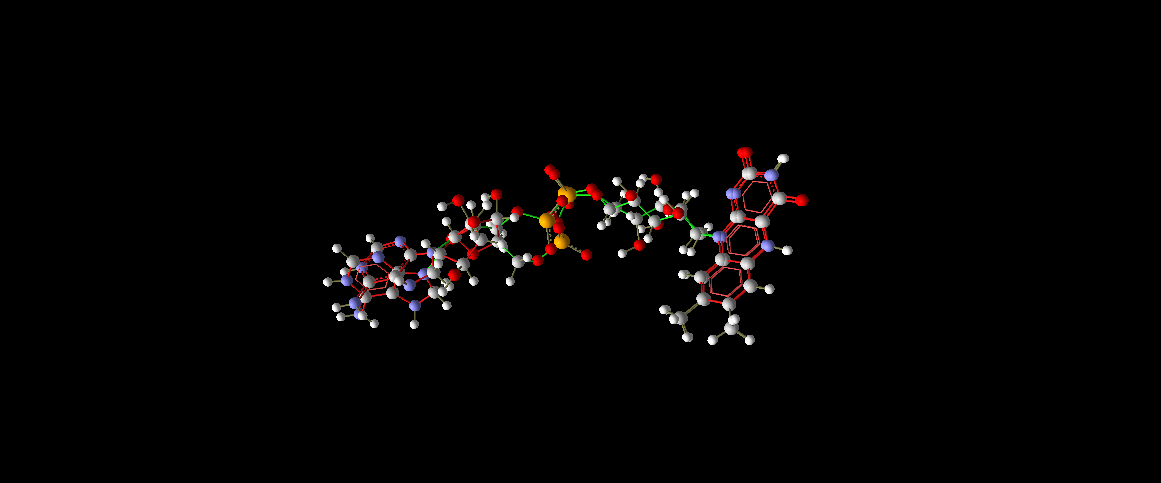


**FIGURE S11** Superimposition of the 4ynt co-crystallized ligand and the docked pose of the same ligand, RMSD = 1.546 Å


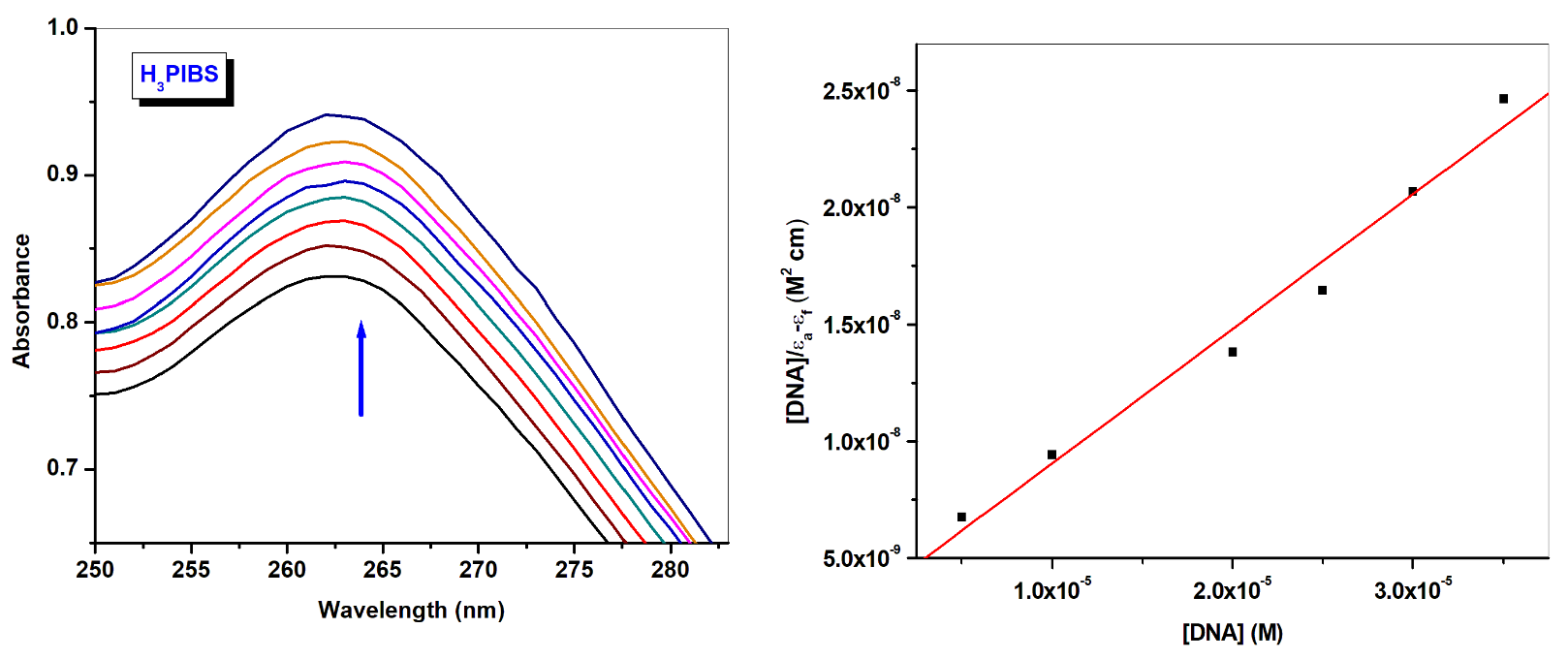


**FIGURE S12** Left: Absorption spectra of constant concentrations of **H_3_PIBS** with different concentrations of SS DNA. Right: Plot of [DNA] vs [DNA] / (εf − εa)


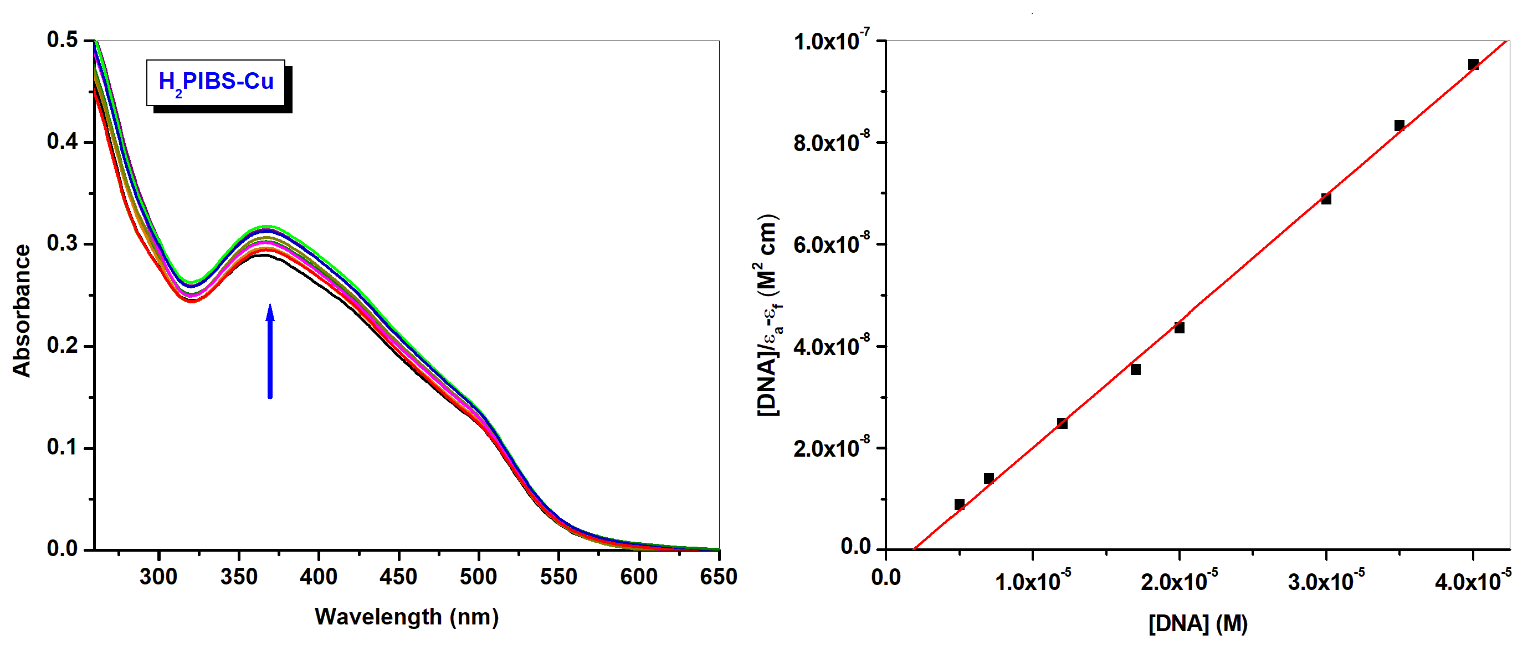


**FIGURE S13** Left: Absorption spectra of constant concentrations of **H_2_PIBS-Cu** with different concentrations of SS DNA. Right: Plot of [DNA] vs [DNA] / (εf − εa)


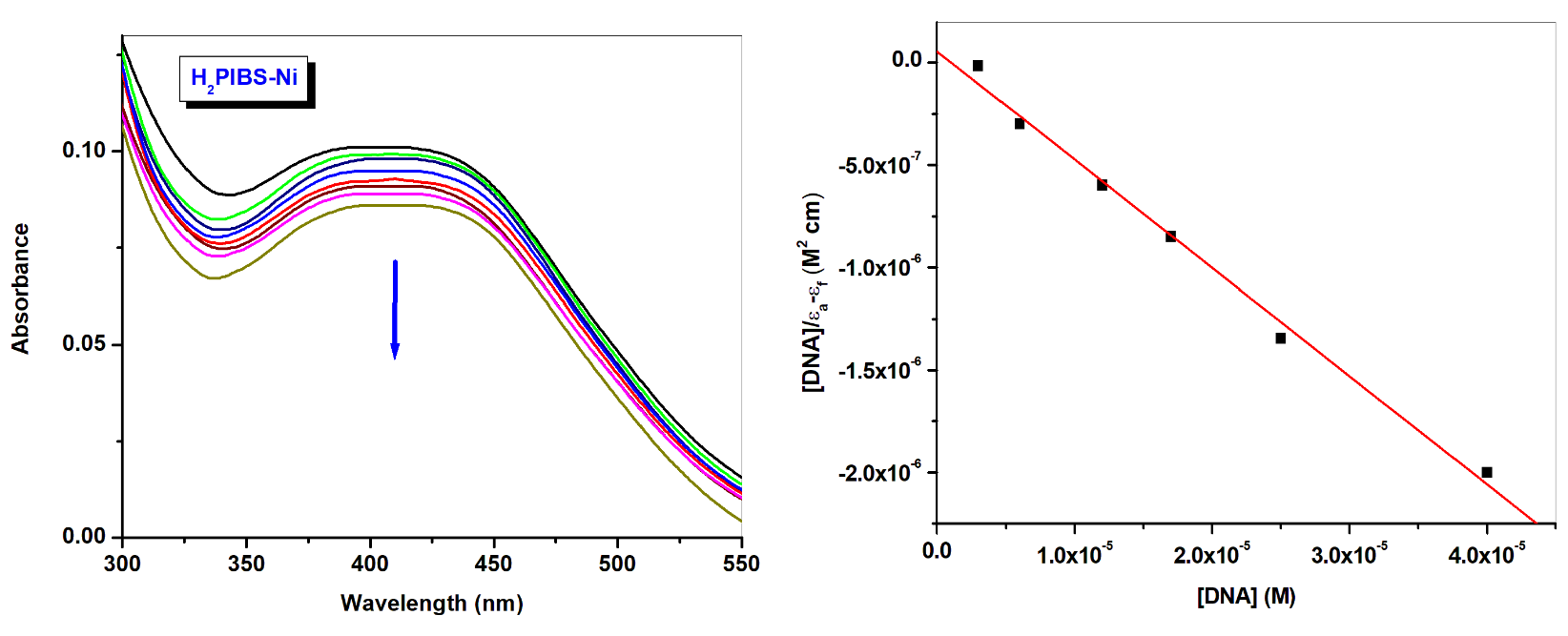


**FIGURE S14** Left: Absorption spectra of constant concentrations of **H_2_PIBS-Ni** with different concentrations of SS DNA. Right: Plot of [DNA] vs [DNA] / (εf − εa)


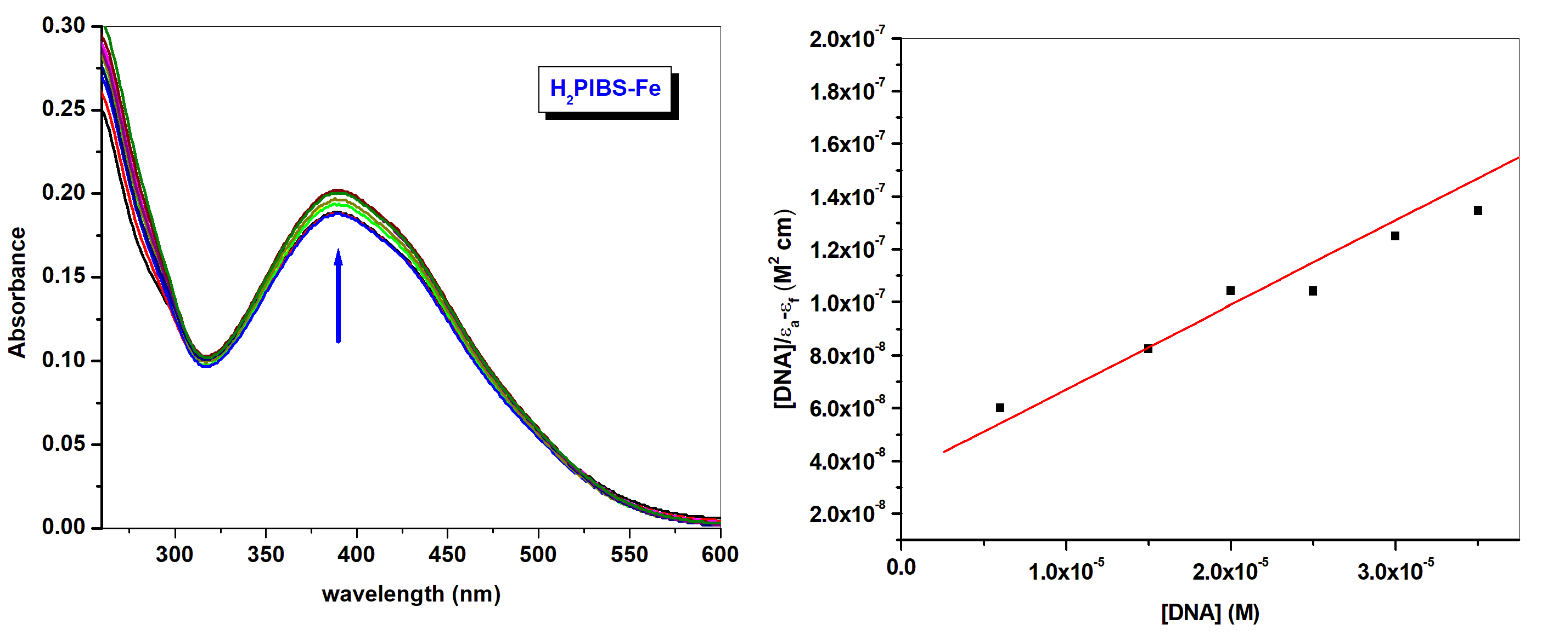


**FIGURE S15** Left: Absorption spectra of constant concentrations of **H_2_PIBS-Fe** with different concentrations of SS DNA. Right: Plot of [DNA] vs [DNA] / (εf − εa)

**Table S1** The *in-vitro* antitumor activity (IC_50_) of the **H_3_PIBS** and its complexes against A-549 and **PANC-1** cell line

| **Compound** | **IC_50_ (µg/ml)** | |
| --- | --- | --- |
|  | **A-549** | **PANC-1** |
| **H_3_PIBS** | 466.25± 17.52 | 360.61 ± 14.63 |
| **H_2_PIBS-Cu** | 12.26 ± 0.73 | 13.43 ± 0.81 |
| **H_2_PIBS-Ni** | 176.48± 10.72 | 120.84 ± 6.28 |
| **H_2_PIBS-Co** | 248.38 ± 14.65 | 185.81 ± 9.73 |
| **H_2_PIBS-Fe** | 154.73± 11.48 | 102.03 ± 5.95 |
| **H_2_PIBS-Zn** | 137.81± 10.23 | 98.94± 5.78 |
| **Vinblastine Sulfate** | 24.6 ± 0.65 | 4.68 ± 0.65 |

**References**

[1] W. B. Williams, M. E. Cuvelier, C. Berset, Use of a free radical method to evaluate antioxidant activity, *Food Sci. Technol.* **1995**, *28*, 25–30.
